# Supplementary material for: Re-organization of Pacific overturning circulation across the Miocene Climate Optimum
Source: Nat Commun. 2024 Sep 17;15:8135. doi: 10.1038/s41467-024-52516-x (PMC11408672; doi:10.1038/s41467-024-52516-x)
Supplement: Supplementary file 1 — Supplementary Information [file 41467_2024_52516_MOESM1_ESM.pdf]

SUPPLEMENTARY INFORMATION

Re-organization of Pacific overturning circulation across the Miocene Climate Optimum

Ann Holbourn[1], Wolfgang Kuhnt[1], Denise K. Kulhanek[1], Gregory Mountain[2], Yair Rosenthal[2,3], Takuya Sagawa[4], Julia Lübbers[1,5], Nils Andersen[6]

[1]*Institute of Geosciences, Christian-Albrechts-University, D-24118 Kiel, Germany*

[2]*Department of Earth and Planetary Sciences, Rutgers, The State University of New Jersey, Piscataway, NJ, USA*

[3]*Department of Marine and Coastal Sciences, Rutgers, The State University of New Jersey, New Brunswick, NJ, USA*

[4]*Faculty of Geosciences and Civil Engineering, Institute of Science and Engineering, Kanazawa University, Japan*

[5]*Center for Marine and Environmental Research (CIMA), University of Algarve, Faro, Portugal*

[6]*Leibniz Laboratory for Radiometric Dating and Stable Isotope Research, Christian-Albrechts-University Kiel, D-24118, Kiel, Germany*

Corresponding author: Ann Holbourn; email: ann.holbourn@ifg.uni-kiel.de

SUPPLEMENTARY NOTE 1: *Modern Pacific overturning circulation*

SUPPLEMENTARY NOTE 2: *Composite depth scale and chronology*

SUPPLEMENTARY NOTE 3: *Benthic foraminifer stable isotopes: preservation, interspecies offsets and spectral characteristics*

SUPPLEMENTARY NOTE 4: *Carbonate weight % estimates from calibrated X-ray fluorescence (XRF) scanner-derived elemental data*

SUPPLEMENTARY NOTE 5: *Carbonate accumulation, dissolution and carbonate ion concentration*

SUPPLEMENTARY NOTE 6: *XRF scanner-derived Log(Si/Ti) as proxy for biogenic silica*

SUPPLEMENTARY NOTE 7: *Comparison of stable isotope records from Pacific and Indian Oceans*

SUPPLEMENTARY NOTE 1

Modern Pacific overturning circulation

The modern Pacific deep water circulation is dominated by the influx of deep water from the Southern Ocean along the western Pacific margin from New Zealand to the Philippines (Antarctic Bottom Water, AABW and Circumpolar Deep Water CDW)[1-3]. This water mass rises through diapycnal diffusion in the tropics and subtropics to form Pacific Central Water (PCW), which becomes entrained into the North Pacific anticyclonic gyre and eventually forms the southward return flow of PCW (Figure S1-1).

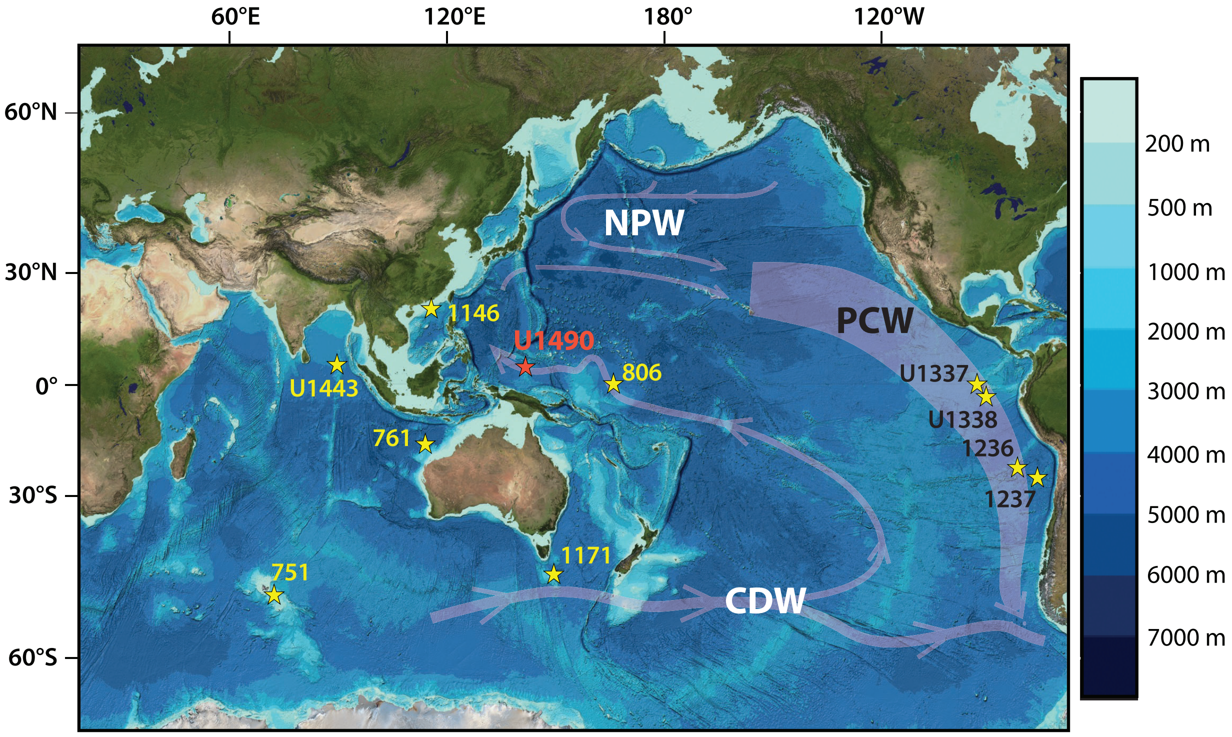

Figure S1-1. Simplified modern Pacific deep water circulation at ~2000 m water depth with locations of Site U1490 and other sites discussed in this study. Arrows indicate main circulation paths of Circumpolar Deep Water (CDW), which rises with Antarctic Bottom Water (AABW) through diapycnal diffusion in the tropics and subtropics and mixes with North Pacific water (NPW) to form Pacific Central Water (PCW). Pacific Central Water represents the main water mass in the central and eastern Pacific Ocean and also forms the return flow to the Southern Ocean[1-2]. Bathymetry is based on gridded bathymetric data sets from the General Bathymetric Chart of the Oceans (GEBCO Compilation Group, GEBCO 2024 Grid (doi:10.5285/1c44ce99-0a0d-5f4f-e063-7086abc0ea0f); land topography is from the Blue Marble satellite mosaic, courtesy of NASA's Earth Observatory (https://neo.gsfc.nasa.gov/view.php?datasetId=BlueMarbleNG-TB).

This circulation pattern results in distinct zonal (Figure S1-2) and meridional (Figure S1-3) gradients. While zonal gradients are weak in the North Pacific, the South Pacific displays a distinct contrast between well ventilated southern-sourced deep waters on the western side and a well-mixed, homogenous, nutrient rich water mass on the eastern side (Figure S1-2). These differences are also reflected in meridional profiles, which show southward extension of PCW into the eastern Pacific and northward extension of Southern Ocean derived water masses into the western Pacific (Figure S1-3a). These patterns affect oxygen contents through the Pacific Ocean as well as the distribution of $\delta^{13}C$, alkalinity and dissolved silicon (Figure S1-3b).

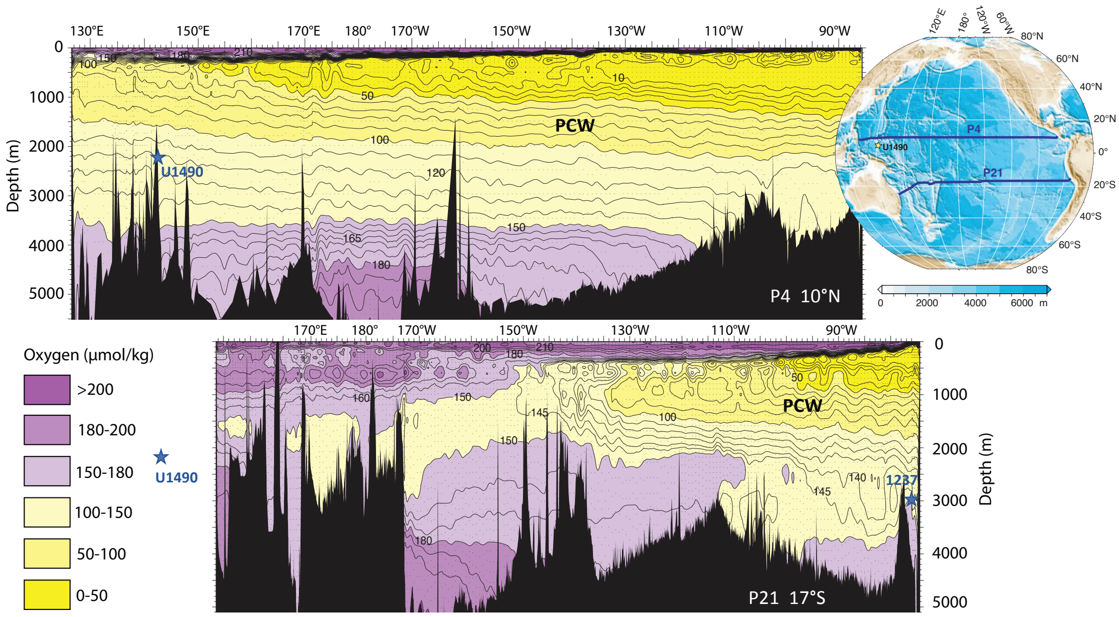

Figure S1-2. Oxygen concentration and water mass distribution along E-W zonal transects at 10°N and 17°S from WOCE Pacific Ocean Atlas[4]. The influence of less ventilated Pacific Central Water (PCW) is restricted to the eastern tropical Pacific across the Southern Hemisphere transect at 17°S (upper panel), whereas the influence of PCW reaches the western tropical Pacific across the northern transect at 10°N (lower panel).

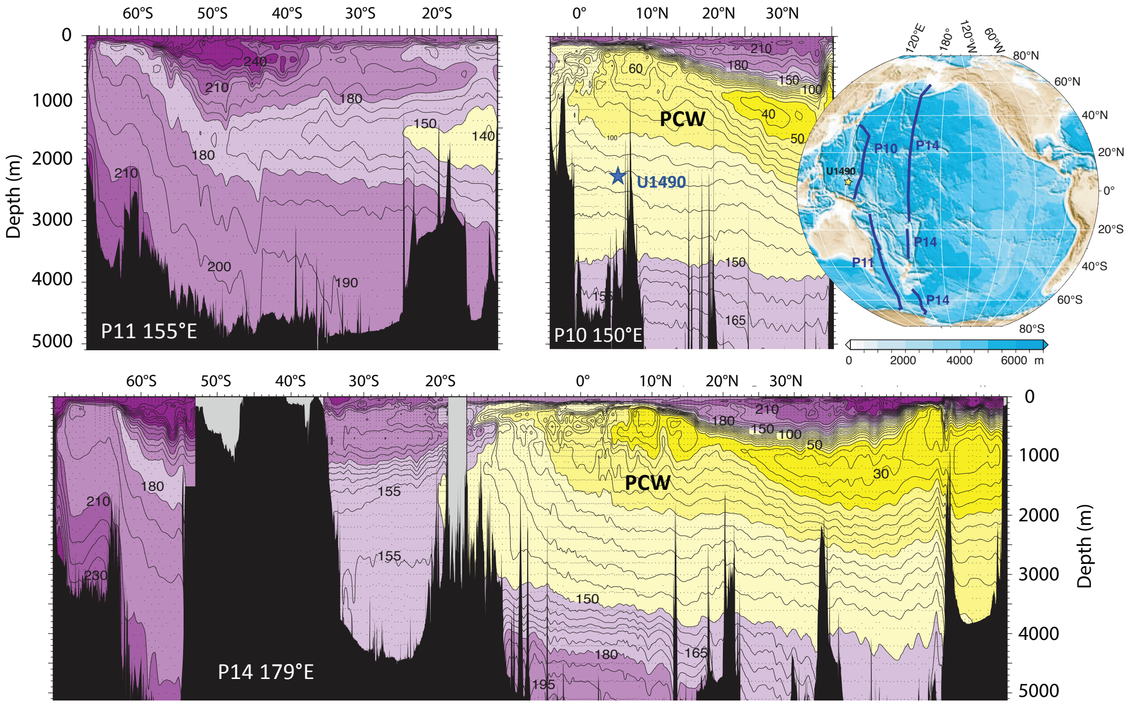

Figure S1-3a. Oxygen concentration and water mass distribution along N-S meridional transects in the western Pacific at 155°E (upper panel left), 150°E (upper panel right) and 179°E (lower panel) from the WOCE Pacific Ocean Atlas[4]. Less ventilated Pacific Central Water (PCW) influences the western tropical Pacific to ~5°N at 150°E and to ~5°S at 179°E.

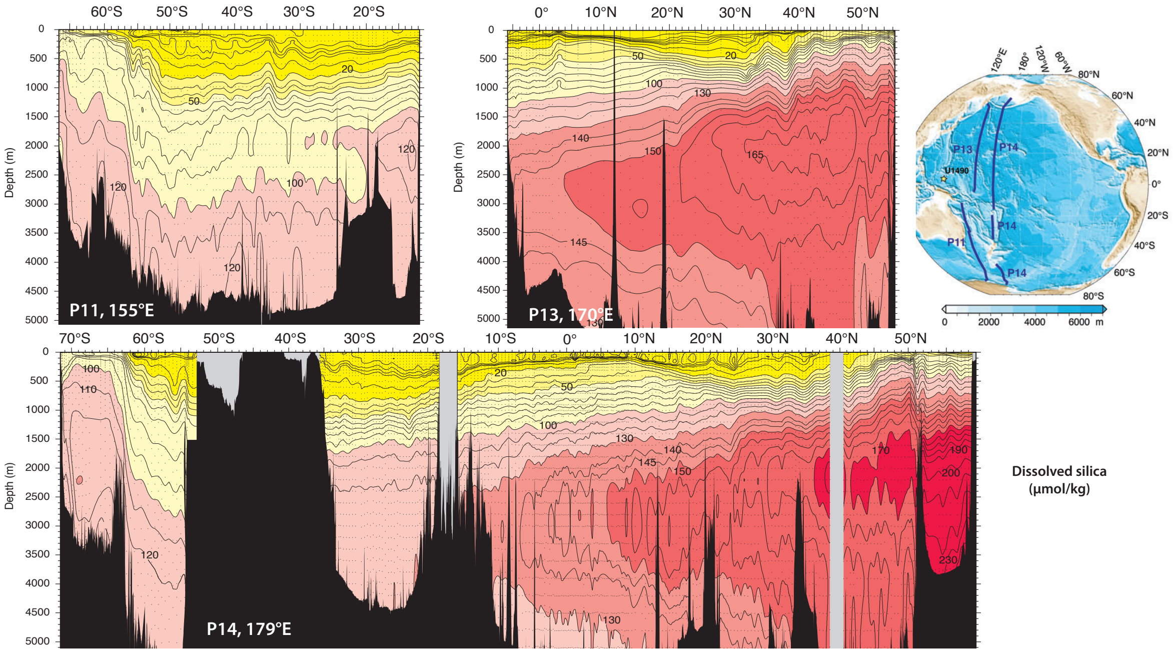

Figure S1-3b. Dissolved silica concentration along N-S meridional transects in the western Pacific at 155°E (upper panel left), 170°E (upper panel right) and 179°E (lower panel) from the WOCE Pacific Ocean Atlas[4].

SUPPLEMENTARY NOTE 2

Site U1490 revised composite depth scale and stratigraphic splice

We used XRF core scanning measurements combined with physical property data (magnetic susceptibility [MS], gamma ray attenuation [GRA] bulk density, natural gamma radiation [NGR], and color reflectance [L*, a*]) to improve the shipboard composite depth scale and stratigraphic splice for Site U1490[5]. Details of the XRF core scanning procedure are provided in "Methods". All sections included in the original shipboard splice were scanned, together with additional adjacent sections, when necessary, to provide sufficient overlap at tie points in order to confirm the ties. X-ray

fluorescence scanner data, together with line-scan core images and physical property data, were loaded into Igor Pro (Wavemetrics) using the Code for Ocean Drilling Data (CODD) of ref. 6 to facilitate checking and updating of the shipboard composite depth scale and stratigraphic splice. Whenever possible, the original tie point was retained in one hole and the hole tied to it adjusted to provide a better fit of the data. In a few instances, the splice tie point was moved to avoid including disturbed intervals in the stratigraphic splice. Core images and physical property data are archived in the IODP Laboratory Information Management System (LIMS) http://web.iodp.tamu.edu/LORE/.

In total, 28 tie points were adjusted in one hole using XRF data (Ca/(Al+K+Ti+Fe) and Ba/Sr) and physical property data to provide a better fit of data. Additionally, five tie points were adjusted in both holes to avoid inclusion of potentially disturbed intervals or to move the tie point to a position, where the XRF or physical property data better aligned. Most tie points were only adjusted a few centimeters; however, in a few instances, larger adjustments were made. The dataset that most clearly resolved the overlaps across tie points was used to revise the position of the tie points; however, each revision was also checked in the other datasets to ensure a good match. The adjusted positions of tie points within the interval 18.2 to 12.5 Ma are shown in Table S2-1. Here, we illustrate the two larger adjustments that were made in the interval 18.2 to 12.5 Ma. Figure S2-1 shows the tie from Cores U1490B-25H to U1490A-25H. Shifting the tie point 0.53 m deeper in Hole U1490A resulted in a much better match between the Ba/Sr records. Similarly, we shifted the tie point 0.48 m shallower in Core U1490A-27H, which resulted in a much better match between the Log(Ca/Terr.) records of Cores U1490B-29F and U1490A-27H (Figure S2-2).

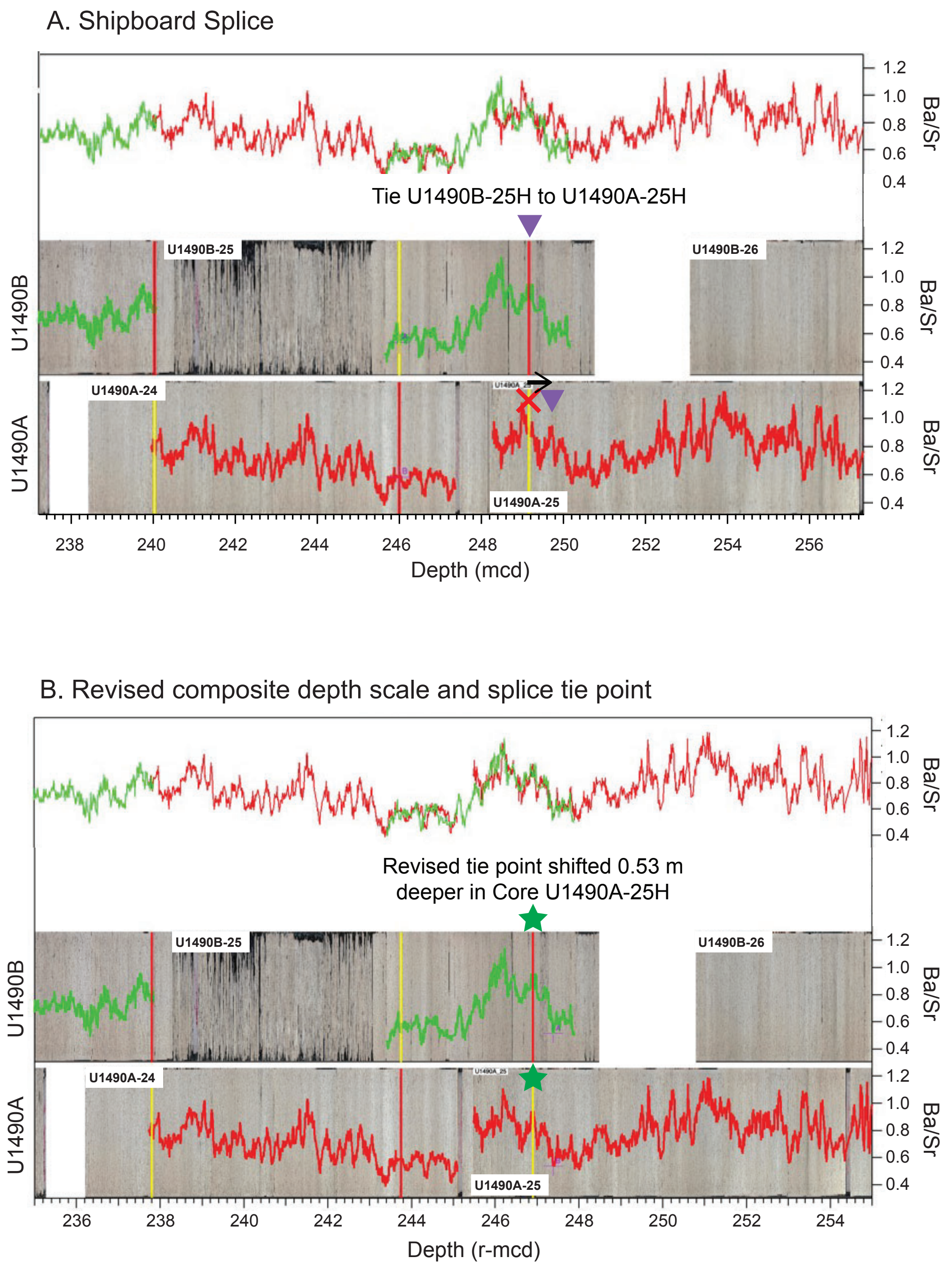

Figure S2-1. Comparison of tie point from Core U1490B-25H to Core U1490A-25H on the original shipboard composite depth scale (mcd) in panel A and the revised composite depth scale (r-mcd) and stratigraphic splice in panel B. Each panel shows Ba/Sr ratio plotted on top of line scan images from Holes U1490A (red) and U1490B (green). The top graph shows the overlay of the Ba/Sr from both holes. Red and yellow lines on core images denote top (yellow) and base (red) of splice intervals. The original shipboard tie point (panel A) was shifted deeper in Core U1490A-25H to provide a better match in the Ba/Sr data (panel B). The green stars in panel B denote the final position of the revised tie point.

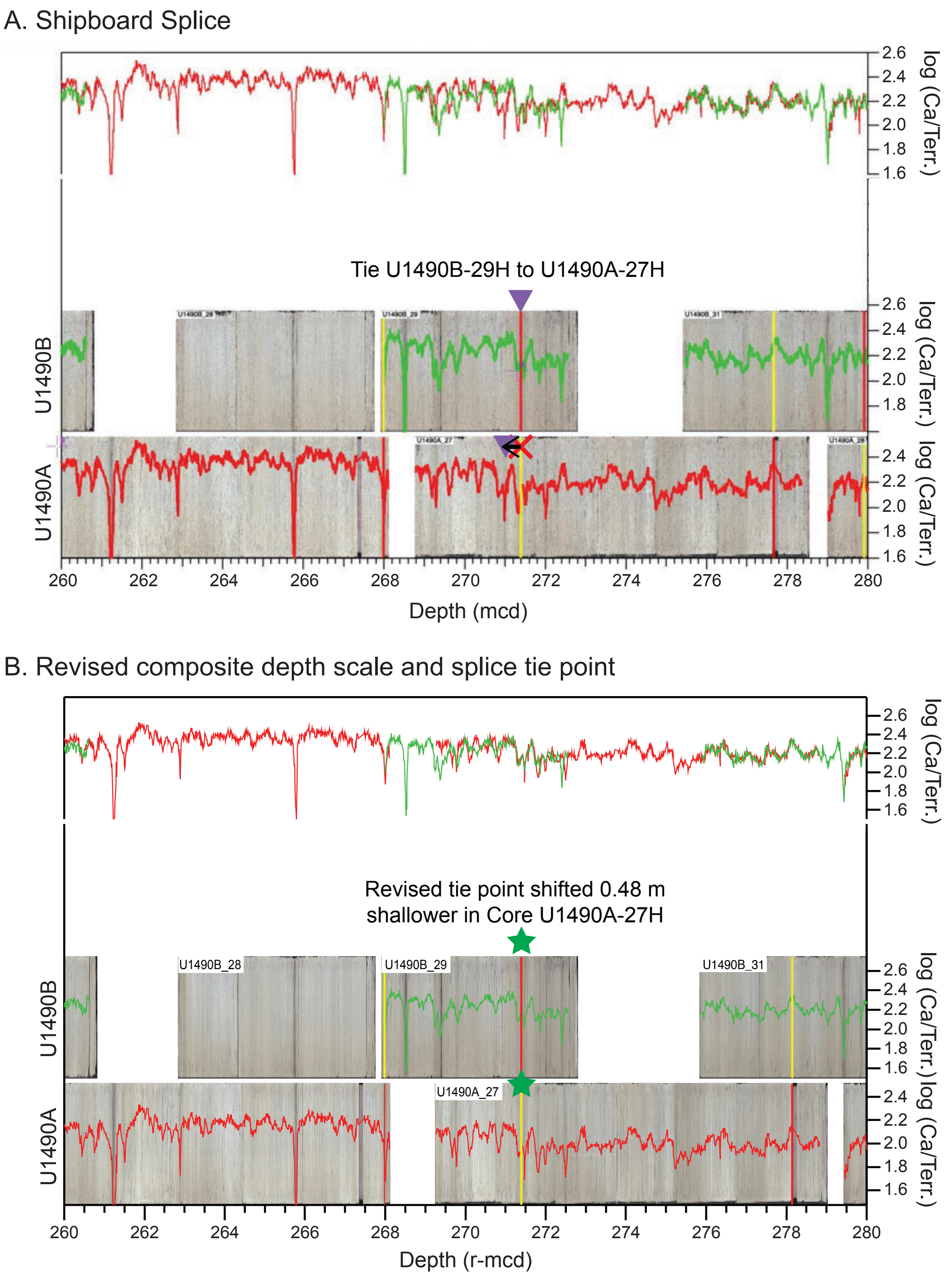

Figure S2-2. Comparison of tie point from Core U1490B-29F to Core U1490A-27H on the original shipboard composite depth scale (mcd) in panel A and the revised composite depth scale (r-mcd) and stratigraphic splice in panel B. Each panel shows Log(Ca/Terr.), where Terr. = Al+K+Ti+Fe, plotted on top of line scan images from Holes U1490A (red) and U1490B (green). The top graph shows the overlay of the Log(Ca/Terr.) from both holes. Red and yellow lines on core images denote top (yellow) and base (red) of splice intervals. The original shipboard tie point (panel A) was shifted deeper in Core U1490A-27H to provide a better match in the Log(Ca/Terr.) data (panel B). The green stars in panel B denote the final position of the revised tie point.

Table S2-1: Adjusted positions of revised splice tie points within the interval 18.2 to 12.5 Ma. (r-mcd = revised composite depth).

Hole	Core	Type	Section	Interval	Depth mbsf	Depth r-mcd	Tie Type	Hole	Core	Type	Section	Interval	Depth mbsf	Depth r-mcd	Comments
U1490 A	23	H	3	138	208.08	229.75	TIE	U1490 B	24	H	1	89	209.89	229.75	Tie point adjusted in B24
U1490 B	24	H	6	144	217.94	237.8	TIE	U1490 A	24	H	2	9	214.79	237.8	Tie point adjusted in A24
U1490 A	24	H	6	3	220.73	243.74	TIE	U 1490 B	25	H	5	35	223.95	243.74	Tie point adjusted in B25
U1490 B	25	H	7	49	227.1	246.89	TIE	U1490 A	25	H	1	141	224.11	246.89	Tie point adjusted in A25
U1490 A	25	H	7	69	232.39	255.17	APPEND	U1490 B	27	F	1	0	232.7	255.93	No change
U1490 B	27	F	3	43	236.13	259.36	TIE	U1490 A	26	H	1	92	233.12	259.36	No change
U1490 A	26	H	7	54	241.74	267.98	TIE	U1490 B	29	F	1	7	242.17	267.98	No change
U1490 B	29	F	3	48	245.58	271.39	TIE	U1490 A	27	H	2	64	243.84	271.39	Tie point adjusted in A27
U1490 A	27	H	6	139	250.59	278.14	TIE	U1490 B	31	F	2	81	251.11	278.14	Tie point adjusted in B31
U1490 B	31	F	4	49	253.29	280.32	TIE	U1490 A	28	F	1	90	252.1	280.32	No change
U1490 A	28	F	2	69	253.39	281.61	TIE	U1490 B	32	F	1	30	253.8	281.61	No change
U1490 B	32	F	3	77	257.27	285.08	TIE	U1490 A	29	F	1	66	256.56	285.08	Tie point adjusted in A29
U1490 A	29	F	2	87	258.27	286.79	TIE	U1490 B	33	F	1	27	258.47	286.79	No change
U1490 B	33	F	4	33	262.54	290.86	TIE	U1490 A	30	F	1	150	262.1	290.86	No change
U1490 A	30	F	3	119	264.79	293.55									

Chronology

Site U1490 astronomically-tuned isotope chronology

The approximate ages for the tuned interval were initially based on the shipboard biostratigraphy[5]. We correlated 30 benthic foraminifer $\delta^{18}O$ minima to maxima in an eccentricity-tilt composite with equal weight of eccentricity (E) and obliquity (T) in radians to which we added 0.2 of the precession parameter P (ET+0.2P) from the La04 astronomical solution[7] (Figures S2-3 and S2-4; Table S2-2). We tuned $\delta^{18}O$ minima to ET maxima, since we assumed that relatively warm summers during high obliquity would promote ice-sheet melting in Antarctica, whereas cool summers during low obliquity would favour ice-sheet growth, and that a low summer insolation gradient between low and high latitudes during high obliquity would decrease poleward moisture transport, inhibiting ice-sheet build-up[8]. Enhanced high-latitude precessional summer insolation during eccentricity maxima would additionally favour the formation of warmer deep water at high latitudes and promote Antarctic ice melt, thus, increasing transfer of ^{16}O from the Antarctic ice sheet to the ocean.

Tuning and filtering were carried out with AnalySeries 2.08[9] using the Linage tuning option and Gaussian filters centered at 0.00247 (405 kyr), 0.01 (100 kyr) and 0.0243 (41 kyr) with bandwidths of 0.0007, 0.003 and 0.005 (Figure S2-4). Intervals of low amplitude 100 kyr eccentricity variability correspond to intervals of low amplitude $\delta^{18}O$ variability between 16.9 and 17.4 Ma and between 14.7 and 14.1 Ma (blue shading in Figure S2-4). Our tuning preserved spectral characteristics evident in the depth domain and minimized changes in sedimentation rates. We compared (1) the filtered $\delta^{13}C$ time series with the 405 kyr component of eccentricity and (2) the 41 and 100 kyr components of the $\delta^{18}O$ time series with filtered obliquity and eccentricity (Figure S2-4). We note, however, that the match of the 41 and 100 kyr filtered $\delta^{18}O$ record with filtered obliquity and eccentricity is ambiguous prior to ~17 Ma.

The onset and development of the warmest phase of the MCO (~16.9-16 Ma) as well as the Mi2 $\delta^{18}O$ maximum[10-12], which terminated this period of peak warmth at ~16 Ma, are better expressed at Site U1490 than at eastern equatorial Pacific Site U1337, enabling an improved correlation to the astronomical solution[7] (Figure S2-4). We therefore slightly modified the Site U1337 tuning of refs. 13-14 by introducing an additional tie point of the $\delta^{18}O$ minimum at 371.61 m to ETP maximum 1670 (Table S2-2).

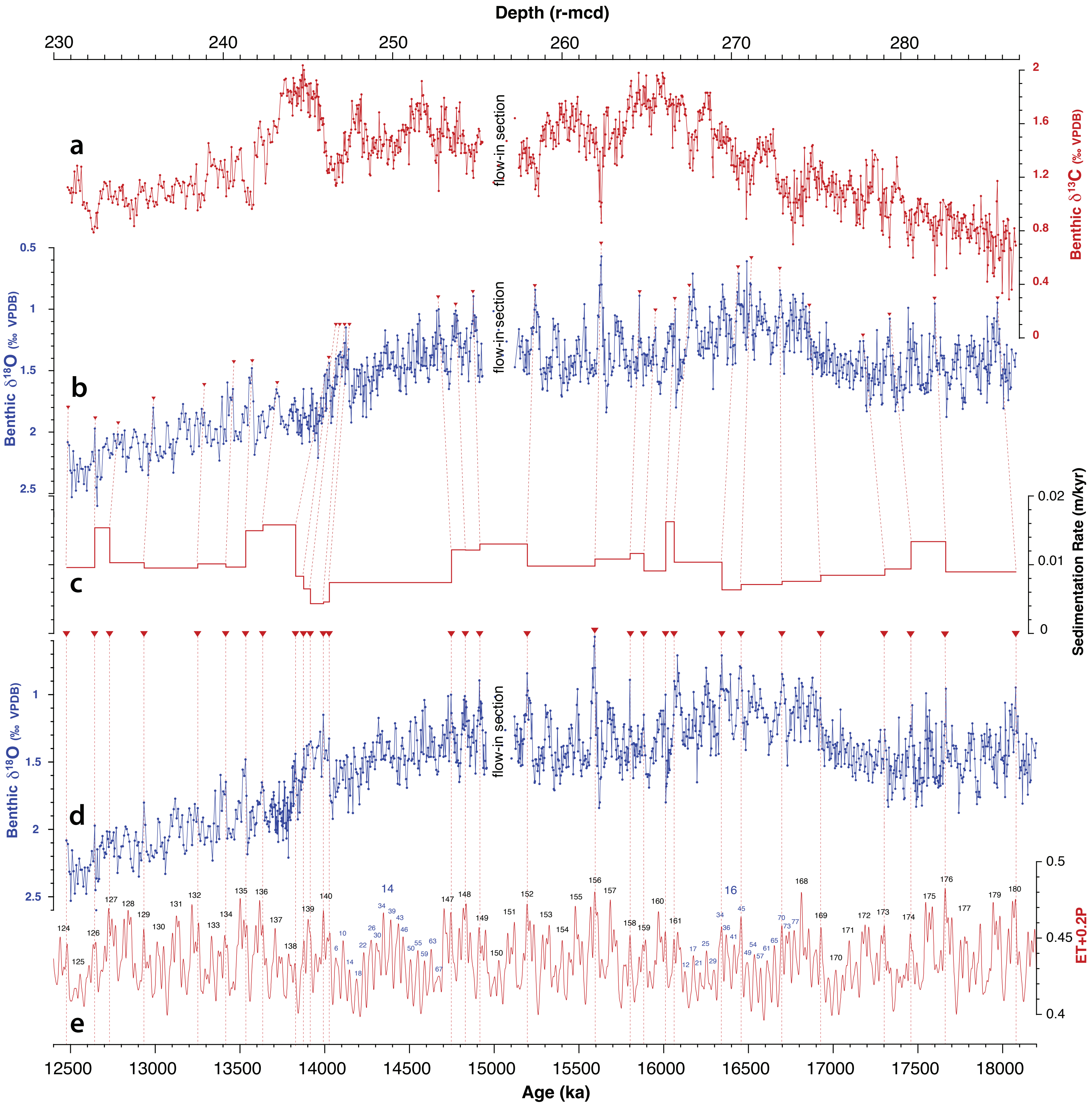

Figure S2-3. Site U1490 benthic foraminifer isotope records between 18.2 and 12.5 Ma (Supplementary Data Files 1-3). **a** Benthic foraminifer $\delta^{13}C$ on revised composite depth scale (meters composite depth: r-mcd). **b** Benthic foraminifer $\delta^{18}O$ on revised composite depth scale. **c** Sedimentation rate. **d** Benthic foraminifer $\delta^{18}O$ on astronomically-tuned timescale. **e** Astronomical tuning target ET+0.2P (ref. 7) with numbering scheme of ref. 14. Tuning tie points to ET+0.2P are marked by red triangles and dashed lines.

Figure S2-4 (next page). Site U1490 astronomically-tuned chronology between 18.2 and 12.5 Ma (Supplementary Data Files 1-3). **a** Benthic foraminifer $\delta^{13}C$ (3 point moving average). **b** 405 kyr filtered eccentricity and 405 kyr filtered $\delta^{13}C$. **c** Wavelet power spectrum of benthic foraminifer $\delta^{18}O$. Black line indicates cone of influence; dotted lines indicate 41, 100, 400 kyr periods. **d** Benthic foraminifer $\delta^{18}O$ and eccentricity 100 kyr filtered. **e** Benthic foraminifer $\delta^{18}O$ and obliquity 41 kyr filtered. **f** Sedimentation rate. **g** Benthic foraminifer $\delta^{18}O$ (3 point moving average). **h** Astronomical tuning target ET+0.2P (ref. 7) with numbering scheme of ref. 14. All isotope data are given in ‰ vs. VPDB. Tuning tie points to ET+0.2P are marked by red triangles and dashed lines. Blue shading indicates intervals of weak 100 kyr eccentricity variability and dominant obliquity forcing.

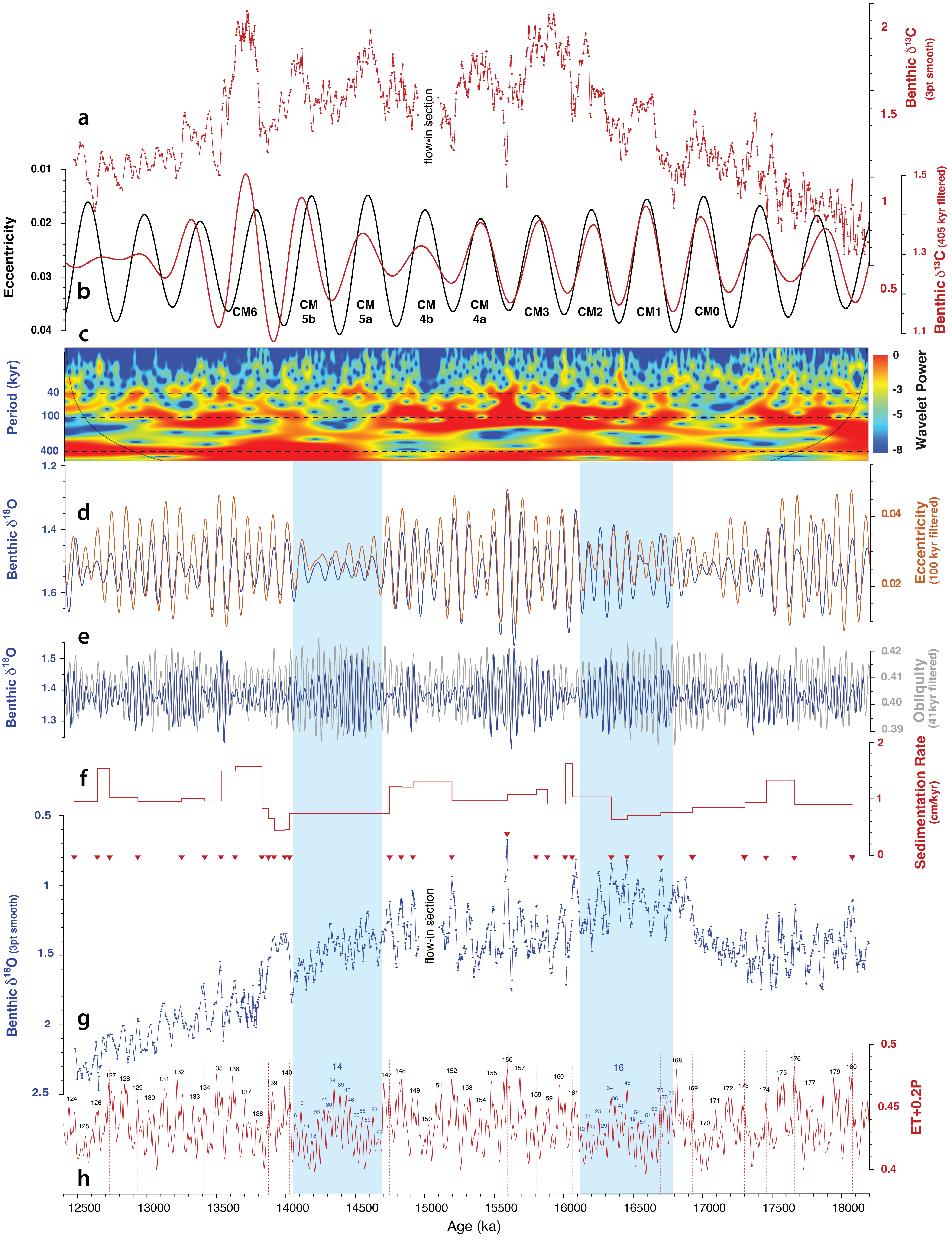

a
b
c
d
e
f
g
h
Benthic δ13C (3pt smooth)
Benthic δ13C (405 kyr filtered)
Eccentricity
Period (kyr)
Wavelet Power
Benthic δ18O
Eccentricity (100 kyr filtered)
Obliquity (41kyr filtered)
Sedimentation Rate (cm/kyr)
Benthic δ18O (3pt smooth)
ET+0.2P
flow-in section
CM6
CM 5b
CM 5a
CM 4b
CM 4a
CM3
CM2
CM1
CM0
Age (ka)

Table S2-2: Site U1490 $\delta^{18}O$ tie points to ET+0.2P from the La04 astronomical solution[7], mbsf = meters below seafloor, mcd= meters composite depth, r-mcd = revised composite depth.

Hole-Core-Section, depth(cm)	mbsf	mcd	r-mcd	Age (ka)	ET+0.2P maximum
U1490B - 24 H - 2, 46	210.96	233.05	230.82	12476	124
U1490B - 24 H - 3, 56	212.56	234.65	232.42	12642	126
U1490B - 24 H - 4, 42	213.92	236.01	233.78	12731	127
U1490B - 24 H - 5, 100	216.00	238.09	235.86	12933	129
U1490A - 24 H - 2, 118	215.88	241.06	238.89	13250	132
U1490A - 24 H - 3, 136	217.56	242.74	240.57	13416	134
U1490A - 24 H - 4, 98	218.68	243.86	241.69	13533	135
U1490A - 24 H - 5, 98	220.18	245.36	243.19	13634	136
U1490B - 25 H - 6 136	226.46	248.57	246.25	13827	138
U1490B - 25 H - 7, 24	226.85	248.96	246.64	13875	139a
U1490A - 25 H - 1, 144	224.14	249.76	246.92	13915	139b
U1490A - 25 H - 2, 26	224.46	250.08	247.24	13991	140a
U1490A - 25 H - 2, 42	224.62	250.24	247.40	14026	140b
U1490A - 25 H - 5, 124	229.94	255.56	252.72	14746	147
U1490A - 25 H - 6, 76	230.96	256.58	253.74	14829	148
U1490A - 25 H -7, 28	231.98	257.60	254.76	14914	149
U1490B - 27 F - 2, 98	235.18	261.24	258.41	15194	152
U1490A - 26 H - 3, 88	236.08	265.16	262.32	15593	156
U1490A - 26 H - 5, 12	238.32	267.40	264.56	15800	158
U1490A - 26 H - 5, 108	239.28	268.36	265.52	15893	159
U1490A - 26 H - 6, 74	240.44	269.52	266.68	16010	160
U1490A - 26 H - 7, 8	241.28	270.36	267.52	16061	161
U1490B - 29 F - 2, 102	244.62	273.27	270.43	16343	1634
U1490B - 29 F - 3, 24	245.34	273.99	271.15	16455	1645
U1490A - 27 H - 3, 62	245.32	275.24	272.87	16698	1670
U1490A - 27 H - 4, 84	247.04	276.95	274.59	16926	169
U1490A - 27 H - 5, 18	247.88	277.80	275.43	17304	173
U1490B - 31 F - 3, 40	252.20	281.65	279.23	17458	174
U1490B - 32 F - 1, 68	254.18	284.41	281.99	17663	176
U1490A - 29 F - 1, 128	257.18	288.09	285.70	18077	180

Chronology of ODP and IODP sites discussed in this work

Sites U1337-U1138: The combined benthic foraminifer stable isotope record of Sites U1337-U1338 versus ages and revised composite depth scales[13-15] is provided in Supplementary Data File 6. We introduced an additional tie point in the U1337 age model at 371.61 revised mcd (r-mcd), which corresponds to the ETP maximum at 16698 ka. We define the tie point between the U1337 and U1338 records at 15.974 Ma between Sample 321-U1338C-44H-6, 60-62 cm (395.29 mbsf, 435.60 mcd, 436.40 m revised mcd (r-mcd); $\delta^{18}O$ = 1.40‰, $\delta^{13}C$ = 1.73‰, 15.971 Ma) and Sample 321-U1337C-20X-3, 94-96 cm (321.24 mbsf, 358.31 r-mcd; $\delta^{18}O$ = 1.78‰, $\delta^{13}C$ = 1.55‰, 15.976 Ma). The revised U1337 record is based on 28 tie points, as shown in Table S2-3 (the additional tie point is marked in bold italics).

Table S2-3: Site U1337 $\delta^{18}O$ tie points to ET+0.2P from the La04 astronomical solution[7]

Hole-Core-Section, depth(cm)	mbsf	mcd	r-mcd	Age (ka)
321-U1337C-18X-2,82	300.42	335.46	335.46	14949
321-U1337C-18X-4,52	303.12	338.16	338.16	15080
321-U1337C-18X-4,112	303.72	338.76	338.76	15119
321-U1337C-18X-5,82	304.92	339.96	339.96	15194
321-U1337C-18X-6,12	305.72	340.76	340.76	15234
321-U1337A-34X-3,32	312.62	341.52	341.52	15286
321-U1337A-34X-3,132	313.62	342.52	342.52	15325
321-U1337A-34X-5,52	315.82	344.72	344.72	15402
321-U1337C-19X-4,7	312.27	347.3	347.3	15479
321-U1337A-35X-2,82	321.22	349.87	349.87	15570
321-U1337A-35X-3,17	322.07	350.73	350.73	15593
321-U1337D-36X-4,22	319.22	353.21	353.21	15720
321-U1337D-36X-4,142	320.42	354.41	354.41	15800
321-U1337C-20X-3,82	321.12	356.79	358.19	15970
321-U1337C-20X-4,144	323.24	358.8	360.3	16083
321-U1337C-21X-1,127	328.07	365.07	365.97	16341
321-U1337C-21X-2,131	329.61	366.61	367.51	16456
321-U1337C-21X-5,89	***333.69***	***370.71***	***371.61***	***16698***
321-U1337A-37X-5,44	344.44	375.27	376.17	16927
321-U1337C-22X-3,41	339.81	378.13	379.03	17095
321-U1337C-22X-4,82	341.72	380.04	380.94	17189
321-U1337D-39X-4,32	348.02	384.1	385	17301
321-U1337C-23X-2,13	347.63	387.58	388.48	17455
321-U1337C-23X-4,62	351.12	391.07	391.97	17585
321-U1337D-40X-2,122	355.52	392.28	393.18	17660
321-U1337D-40X-3,52	356.32	393.08	393.98	17728
321-U1337D-40X-4,102	358.32	395.08	395.98	17884
321-U1337D-40X-5,102	359.82	396.58	397.48	17984

Site U1443: Benthic foraminifer stable isotope record on age model of refs. 13-14,16. The isotope record of Site U1443 is provided in Supplementary Data File 13.

Sites 1236 and 1237: Benthic foraminifer stable isotope records of refs. 17-18 on revised age models of refs. 13-14. The isotope record of Sites 1236 and 1237 is provided in Supplementary Data Files 11 and 12.

Hole 1171C: Benthic foraminifer stable isotope record of refs. 19-20 on revised age model, based on correlation to the U1337-U1338 and U1490 records (Table S2-4). The isotope record of Hole 1171C on revised age model is provided in Supplementary Data File 14.

Table S2-4: Site 1171 tie points to the U1337-U1338 and U1490 records

Hole	Core		Section	depth (cm)	mbsf	Age (ka)
1171C	17	X	1	0	144.10	12776
1171C	17	X	4	135	150.00	13036
1171C	18	X	1	40	154.10	13217
1171C	18	X	3	8	156.80	13411
1171C	18	X	3	110	157.80	13502
1171C	18	X	4	120	159.40	13612
1171C	18	X	5	90	160.60	13706
1171C	19	X	1	72	164.00	13874
1171C	19	X	4	24	168.00	14027
1171C	19	X	5	30	169.60	14110
1171C	19	X	6	22	171.00	14190
1171C	19	X	6	110	171.90	14231
1171C	19	X	7	20	172.50	14270
1171C	20	X	4	50	177.90	14588
1171C	22	X	3	30	195.10	15926
1171C	22	X	5	0	197.80	16071

Site 1146: Benthic foraminifer stable isotope record of refs. 13-14, 17 and 21. A splice table, revised age model and stable isotope data versus ages and revised composite depths are provided in Supplementary Data File 7.

Hole 761B: Benthic foraminifer stable isotope record of ref. 22 on revised age model. Recovery at Hole 761B was higher than 100% and to avoid overlap of cores on the mbsf scale, the mbsf depths were converted into a composite depth (mcd) scale[22], as shown below.

Core 761B-4H:
mcd = mbsf, top mbsf 23.2m, top mcd 23.2 m, bottom mbsf 32.7 m, bottom mcd 33.2 m

Core 761B-5H:
mcd = mbsf + 0.50 m; top mbsf 32.7 m, top mcd 33.2 m, bottom mbsf 42.2. m, bottom mcd 43.1 m

Core 761B-6H:
mcd = mbsf + 0.90 m; top mbsf 42.2 m, top mcd 43.1 m, bottom mbsf 51.7 m, bottom mcd 53.3 m

Core 761B-7H:
mcd = mbsf + 160 cm; top mbsf 51.7 m, top mcd 53.3 m, bottom mbsf 61.2 m, bottom mcd 63.3 m

Correlation of the Hole 761B isotope record to the U1337-U1338 and U1490 isotope records was established using 14 tie points, as shown in Table S2-5:

Table S2-5: Site 761 benthic isotope tie points to the U1337-U1338 and U1490 records

Hole-Core-Section, depth(cm)	mbsf	mcd	Age (ka)
761B - 5H - 4, 80	38.00	38.50	12541
761B - 5H - 5, 10	38.80	39.30	12754
761B - 5H - 5, 90	39.60	40.10	13274
761B - 5H - 0, 10	42.30	42.80	14049
761B - 6H - 1, 70	42.90	43.80	14517
761B - 6H - 2, 60	44.30	45.20	15235
761B - 6H - 2, 140	45.10	46.00	15586
761B - 6H - 3, 110	46.30	47.20	15935
761B - 6H - 4, 40	47.10	48.00	16039
761B - 6H - 4, 120	47.90	48.80	16169
761B - 6H- 5, 50	48.70	49.60	16511
761B - 6H- 5, 90	49.10	50.00	16632
761B - 6H - 7, 0	51.20	52.10	16870
761B -7H - 3, 70	55.40	57.00	17383

The isotope record of Hole 761B[22] on revised age model is provided in Supplementary Data File 8.

Hole 751A: Benthic foraminifer stable isotope record of ref. 11 on revised age model of refs. 13-14. The isotope record of Hole 751A is provided in Supplementary Data File 10.

Comparison of astronomically-tuned isotope chronology to Geomagnetic Polarity Time Scales GPTS2012[23] and GPTS2020[24]

Astronomically-tuned ages of magnetochron boundaries agree with GPTS2012[23] ages with two exceptions:

Chrons C5ACn and C5ACr

We attribute the offsets between the astronomically-tuned ages of Chrons C5ACn and C5ACr and the GPTS2012[23] and GPTS2020[24] ages (Table S2-6) to poor definition of chron boundaries in Sections U1490A-25H-1 and -25H-2 and in Core U1490B-25H. In Core U1490B-25H, Chrons C5ABr, C5ABn and C5AARr were not identified, due to a drilling disturbance within the interval U1490B-25H-1, 0 cm to 25H-4, 120 cm. In Sections U1490A-25H-1 and -25H-2 and Sections U1490B-25H-6 and -25H-7, the paleomagnetic signals are poorly resolved and the identification of Chrons C5ACn and C5ACr within these intervals remains questionable.

The U1490 age model between 14.03 and 14.75 Ma is based on a linear interpolation between two tuning tie points that are relatively far apart at 247.40 r-mcd (14.03 Ma) and at 252.73 r-mcd (14.75 Ma) (Table S2-2, Figure S2-3), adding uncertainty to the astronomically-tuned ages of Chrons C5ACn and C5ACr. This interval corresponds to a period of high-amplitude variability in obliquity that is congruent with low variability in short eccentricity. The high-amplitude variability of the obliquity cycle is not, however, clearly imprinted on the benthic $\delta^{18}O$ record, due to the low sedimentation rates within this interval.

Chrons C5Cn.3n to C5Dr.1n

Recent high-precision U-Pb and $^{40}Ar/^{39}Ar$ ages for the onset of Chrons C5Cn.3n to C5Br for the Columbia River Basalt Group (CRBG)[25] agree with the U1490 tuned ages for the base of C5Cn.3n (16651 +32/-31 kyr CRBG age model), C5Cn.2r (16570 +42/-43 kyr CRBG age model), C5Cn2n (16461 +50/-49kyr CRBG age model), C5Cn1r (16370 +56/-60 kyr CRBG age model) and C5Cn.1n (16251 +39/-45 kyr CRBG age model). However, the radiometric age of the base of C5Br (15923 +29/-32 kyr CRBG age model) is 84 kyr younger than our tuned ages.

This discrepancy may be associated with a 60-120 kyr hiatus close to the top of Chron C5Cn.1n, following the main phase emplacement of the CRBG[25].

Astronomically-tuned ages for the base of Chrons C5Dr.1n to C5Cn.3n are consistently younger by 65 to 86 kyr than GPTS2012[23] ages (Figure S2-5, Table S2-6). The first offset downcore at the base of C5Cn.3n at 16.721 Ma is 82 kyr younger than the GPTS2012[23] age (Table S2-6). The close match between the U1490 and U1337 $\delta^{18}O$ and $\delta^{13}C$ records (Figure S2-5) implies that a hiatus/drilling disturbance or splice tie point mismatch at Site U1490 are unlikely causes for this age discrepancy. We note, however, that the ages of magnetic reversals prior to 17 Ma remain tentative, due to ambiguities in correlating the U1490 stable isotope record to the La04 astronomical solution[7].

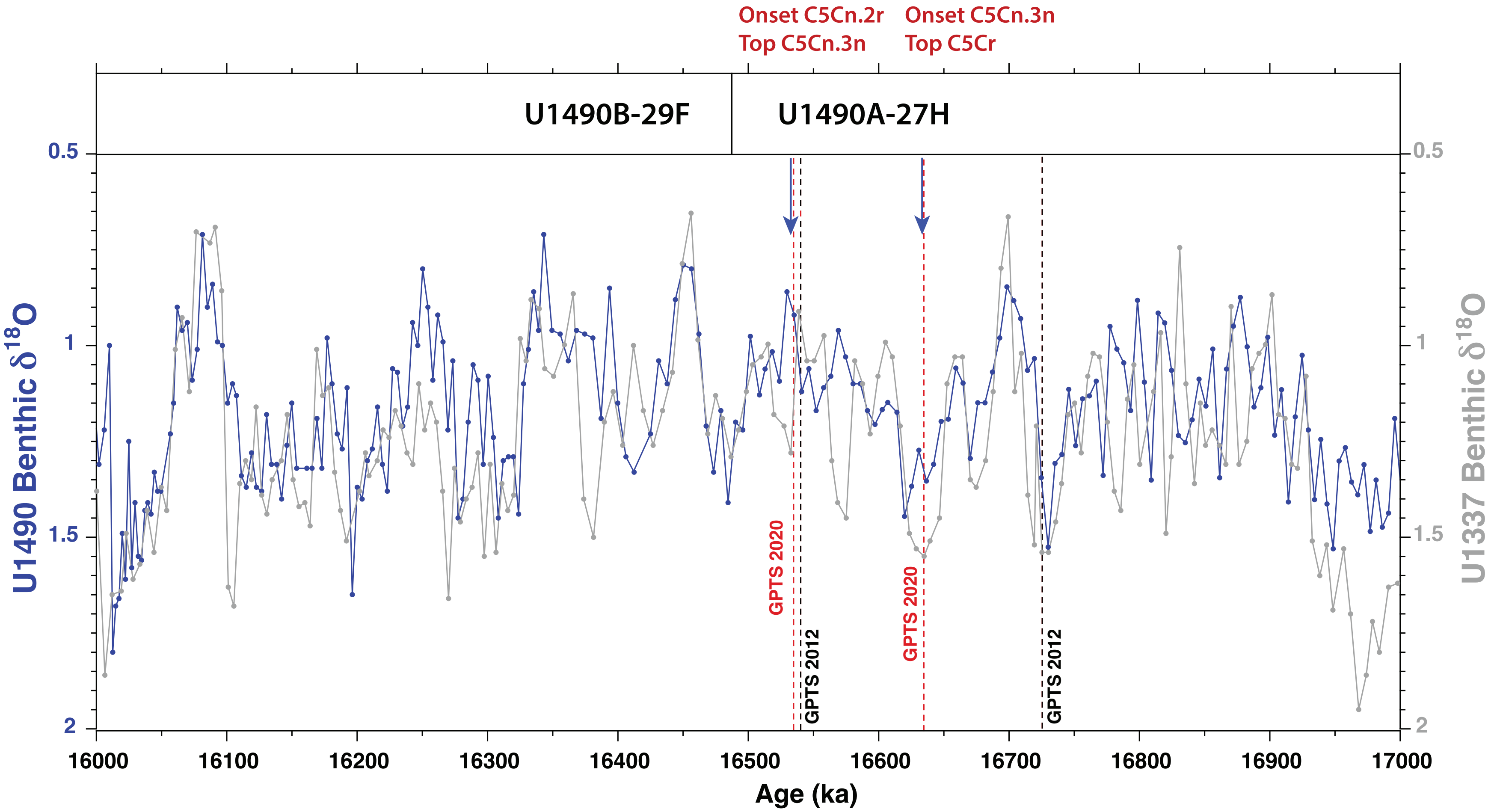

Figure S2-5: Position of Chrons C5Cn.2r and C5n.3n onsets (C5Cn.3n and C5Cr tops) in relation to the benthic foraminifer $\delta^{18}O$ time series at Site U1490 (blue arrows indicate positions of Chrons in Site U1490). Approximately one short eccentricity cycle (~100 kyr) separates the onset of Chrons C5Cn.2r and C5n.3n at Site U1490, in agreement with GPTS2020[24] ages, whereas the time interval should be close to two eccentricity cycles (~180 kyr) according to GPTS2012[23]. The good match between the Sites U1490 and U1337 $\delta^{18}O$ records between 17 and 16 Ma further supports that the GPTS2012[23] age for the base of Chron C5n.3n is too old by ~80 kyr. Dashed red lines indicate GPTS2020[24] ages and dashed black lines GPTS2012[23] ages.

Table S2-6: Astronomically-tuned ages for magnetic polarity Chrons at Site U1490. Deviations of tuned ages from GPTS2012[23], GPTS2020[24] and CRBG ages[25] are marked in red.

Polarity Chron onset	Site	Hole	Core	Type	mid point depth CSF-A (mbsf)	shipboard CCSF-A (mcd)	revised depth (r-mcd)	revised tuned age (ka)	GTS2012 age (ka)	GTS2020 age (ka)	difference to GTS2012 (kyr) revised	difference to GTS2020 (kyr) revised	Kasbohm et al. (2023) age (ka)
**C5Ar.1r**	U1490	B	24	H	213.83	235.92	233.69	12725	12735	12735	-10	-10	
**C5Ar.1n**	U1490	B	24	H	214.48	236.57	234.34	12785	12770	12770	15	15	
**C5Ar.2r**	U1490	B	24	H	215.01	237.10	234.87	12837	12829	12829	8	8	
**C5Ar.2n**	U1490	B	24	H	215.60	237.69	235.46	12894	12887	12887	7	7	
**C5Ar.3r**	U1490	B	24	H	216.91	239.00	236.77	13029	13032	13032	-3	-3	
**C5Ar.3r**	U1490	A	24	H	215.48	240.66	238.49	13209	13183	13183	26	26	
**C5AAr**	U1490	A	24	H	216.90	242.08	239.91	13352	13363	13363	-11	-11	
**C5ABn**	U1490	A	24	H	219.56	244.74	242.57	13592	13608	13608	-16	-16	
**C5ACn**	U1490	B	25	H	226.59	248.69	246.38	13843	14070	14070	-227	-227	
**C5ACr**	U1490	A	25	H	225.14	250.76	247.92	14096	14163	14163	-67	-67	
**C5ADn**	U1490	A	25	H	228.69	254.31	251.47	14576	14609	14609	-33	-33	
**C5ADr**	U1490	A	25	H	230.39	256.01	253.17	14782	14775	14775	7	7	
**C5Bn.1n**	U1490	A	25	H	231.41	257.03	254.19	14866	14870	14870	-4	-4	
**C5Br**	U1490	A	26	H	240.41	269.49	266.65	16007	15974	15994	33	13	15923
**C5Cn.1n**	U1490	B	29	F	243.58	272.23	269.39	16243	16268	16261	-25	-18	16251
**C5Cn.1r**	U1490	B	29	F	244.64	273.29	270.45	16346	16303	16351	43	-5	16370
**C5Cn.2n**	U1490	B	29	F	245.20	273.85	271.01	16434	16472	16434	-38	0	16461
**C5Cn.2r**	U1490	A	27	H	244.14	274.05	271.69	16532	16543	16532	-11	0	16570
**C5Cn.3n**	U1490	A	27	H	244.90	274.81	272.45	16639	16721	16637	-82	2	16651
**C5Cr**	U1490	A	27	H	248.94	278.85	276.49	17149	17235	17154	-86	-5	
**C5Dn**	U1490	B	31	F	252.35	281.80	279.38	17468	17533	17466	-65	2	
**C5Dr.1r**	U1490	A	28	F	253.38	284.02	281.60	17634	17717	17634	-83	0	
**C5Dr.1n**	U1490	B	32	F	254.24	284.47	282.05	17670	17740	17676	-70	-6	
**C5Dr.2r**	U1490	A	29	F	257.08	287.99	285.60	18066	18056	18007	10	59	

SUPPLEMENTARY NOTE 3

Benthic foraminifer stable isotopes: preservation, interspecies offsets and spectral characteristics

Preservation

Benthic foraminifer tests within the upper Lower to Middle Miocene interval (Cores U1490B-29F to U1490A-24H) targeted in this study are overall well preserved, translucent, showing little recrystallisation and minimal infilling (Figure S3-1). In contrast to planktic foraminifer tests, which are generally fragmented over the most of the MCO, the more robust benthic foraminifer tests exhibit only minor signs of carbonate dissolution. However, recrystallization, overgrowth and cementation become severe further downcore below the level of Advanced Piston Corer (APC) and half-length Advanced Piston Corer (HAPC) refusal[5], which corresponds to a lithological change to more indurated sediments.

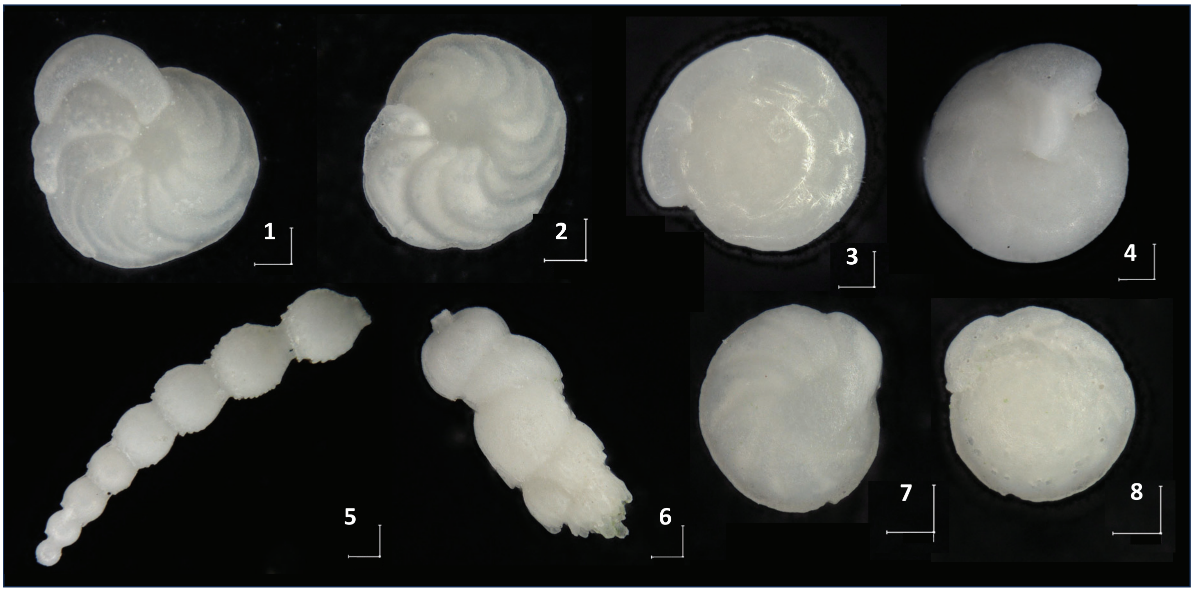

Figure S3-1: Digital light microscope images illustrating typical preservation state of benthic foraminifer taxa selected for stable isotope analysis within the upper Lower to Middle Miocene interval studied at Site U1490. 1. *Cibicidoides wuellerstorfi*, Sample U1490B-25H-5, 86-88 cm, umbilical view (13.70 Ma, 15.9% >63 µm residue weight); 2. *Cibicidoides wuellerstorfi,* Sample U1490B-25H-5, 86-88 cm, umbilical view (13.70 Ma, 15.9% >63 µm residue weight); 3. *Oridorsalis umbonatus,* Sample U1490A-27H-4, 92-94 cm, spiral view (16.93 Ma, 13.3% >63 µm residue weight); 4. *Oridorsalis umbonatus,* Sample U1490A-27H-4, 92-94 cm, umbilical view (16.93 Ma, 13.3% >63 µm residue weight); 5. *Stilostomella subspinosa*, Sample U1490A-27H-2, 147-149 cm (16.61 Ma, 13.8% >63 µm residue weight); 6. *Rectuvigerina* sp., Sample U1490A-27H-3, 6-8 cm (16.62 Ma, 5.9% >63 µm residue weight); 7. *Cibicidoides mundulus,* Sample U1490B-25H-6, 96-98 cm, umbilical view (13.80 Ma, 5.7% >63 µm residue weight); 8. *Cibicidoides mundulus,* Sample U1490B-25H-6, 96-98 cm, spiral view (13.80 Ma, 5.7% >63 µm residue weight). Scale bars equal 100 µm. Note: *Stilostomella subspinosa* was not used for stable isotope analysis, but documents typical preservation state.

Stable isotope interspecies offsets

Site U1490 stable isotope records are provided in Supplementary Data File 1. Stable isotope offsets between *Cibicidoides mundulus* and *Rectuvigerina* sp. and between *Cibicidoides mundulus* and *Oridorsalis umbonatus* are illustrated in Supplementary Figure S3-2 and are listed in Supplementary Data Files 2 and 3.

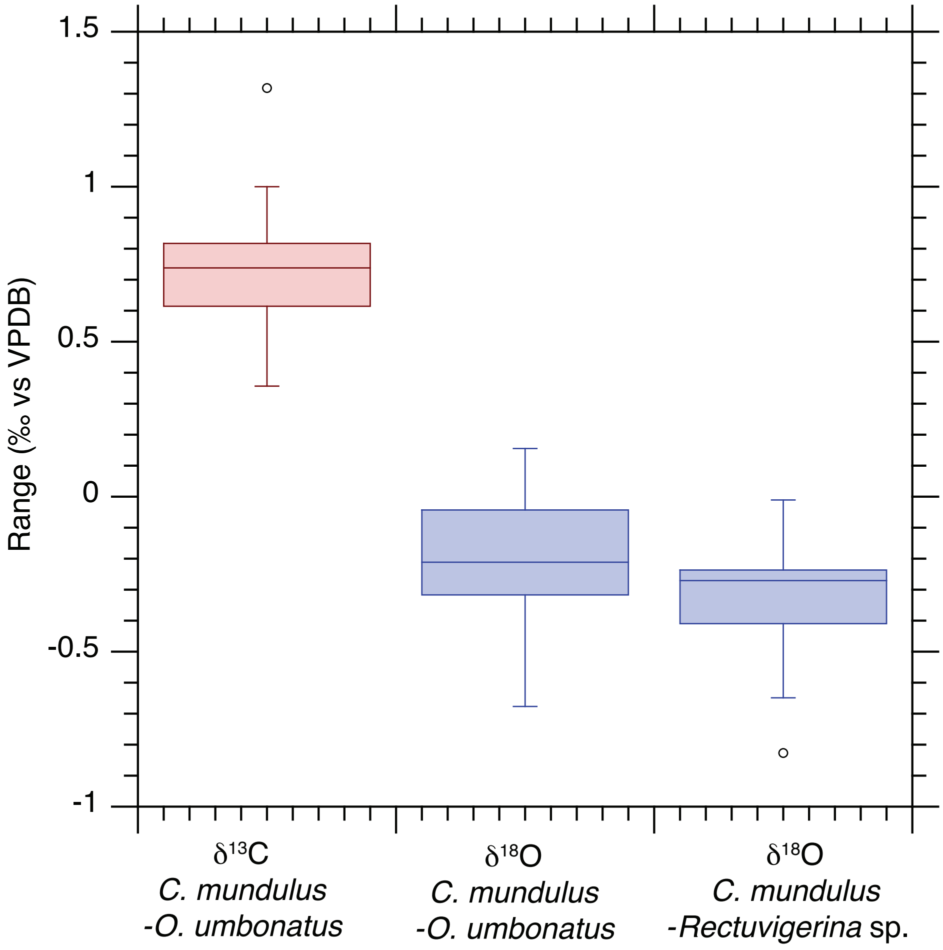

Figure S3-2. Stable isotope offsets between *Cibicidoides mundulus* and *Rectuvigerina* sp. and between *Cibicidoides mundulus* and *Oridorsalis umbonatus*. Each box encloses 50% of the data with the median value (data value in the middle between the lowest and highest value) displayed as a line. The top and bottom of the box marks the limits of the upper and lower 25% (upper and lower quartiles) of the variable population. The lines extending from the top and bottom of each box mark the minimum and maximum values within the data set. The interquartile distance is defined as the difference between the upper and lower quartiles. Outliers (values greater than the upper quartile plus 1.5 times the interquartile distance or smaller than the lower quartile minus 1.5 times the interquartile distance) are displayed as individual points.

Spectral characteristics of stable isotope records

The benthic foraminifer $\delta^{18}O$ time series exhibits a strong response to short eccentricity forcing with distinct peaks at the 124 and 95 kyr periodicities (Figure S3-3), which are typical spectral characteristics of eccentricity[7]. The untuned record in the depth domain also shows the characteristic two spikes of short eccentricity at wavelengths of 1.33 m (>95 % confidence) and 1.04 m (>99 % confidence). The presence of these characteristic periodicities in both time and depths domains demonstrates that the eccentricity signal in the $\delta^{18}O$ record is not artificially induced by tuning.

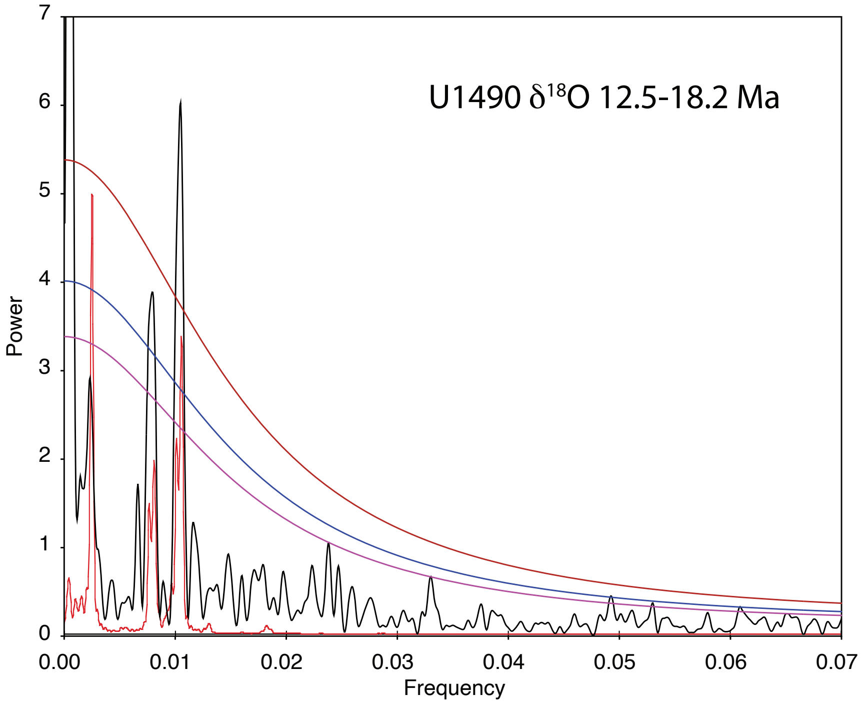

Figure S3-3. Power spectrum of Site U1490 benthic foraminifer $\delta^{18}O$ over the interval 18.2 to 12.5 Ma. REDFIT spectral analysis[26] was performed with PAST 4.10 (ref. 27) using a Hanning window, 6 times oversampling using 3 segments. Confidence intervals (chi^2) of 99 %, 95 % and 90% are given as orange, blue and magenta lines. Blackman-Tukey power spectrum of eccentricity (vertical axis not to scale) for the same time interval is plotted in red for comparison.

A ~400 kyr eccentricity signal dominates in the spectral power of the benthic foraminifer $\delta^{13}C$ record (Figure S3-4). The ~400 kyr cycle in the $\delta^{13}C$ record closely follows the long eccentricity cycle with a phase lag of 28.4 kyr (Figure S3-5). Similar phase lags of ~30 kyr were previously reported from late Early to Middle Miocene high-resolution benthic foraminifer $\delta^{13}C$ records at Site 1146 in the South China Sea and Sites 1237, U1337 and U1338 in the eastern Pacific Ocean[8,14,17].

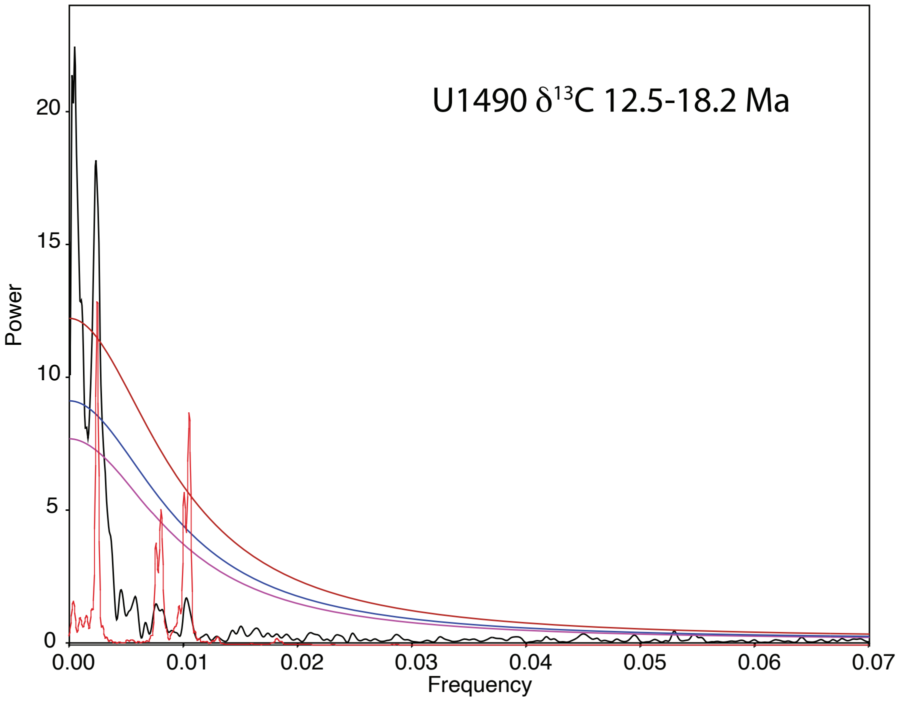

Figure S3-4. Power spectrum of Site U1490 benthic foraminifer $\delta^{13}C$ over the interval 18.2 to 12.5 Ma**.** REDFIT spectral analysis[26] was performed with PAST 4.10 (ref. 27) using a Hanning window, 6 times oversampling using 3 segments. Confidence intervals (chi^2) of 99 %, 95 % and 90 % are given as orange, blue and magenta lines. Note that only the 400 kyr wavelength shows significant spectral power. Blackman-Tukey power spectrum of eccentricity (vertical axis not to scale) for the same time interval is plotted in red for comparison.

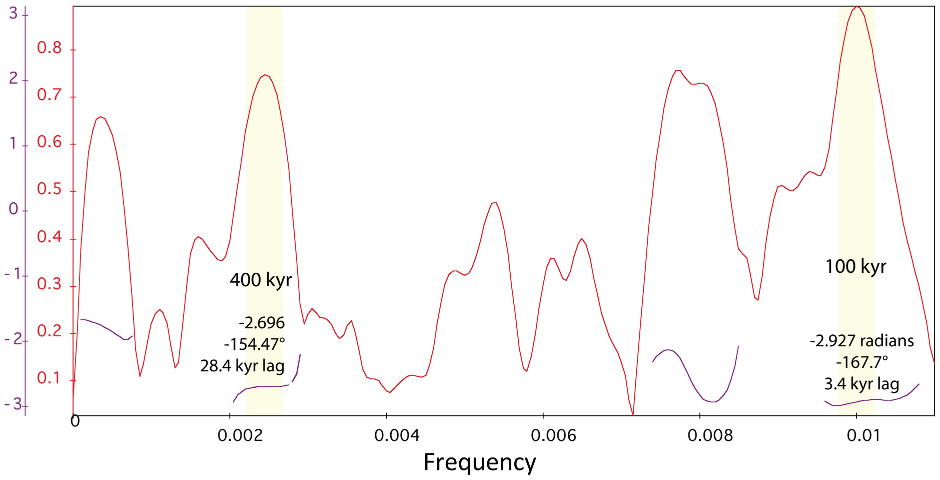

Figure S3-5. Coherence and phase relationship between Site U1490 benthic foraminifer $\delta^{13}C$ and eccentricity over the interval 18.2 to 12.5 Ma. Blackman Tukey cross spectral analysis was performed in AnalySeries 2.08[9] using a Bartlett window with a bandwidth of 0.0008. Non-zero coherence is higher than 0.3. Carbon isotopes are lagging eccentricity by 28.5 kyr at the 400 kyr band and by 3.4 kyr at the 100 kyr band.

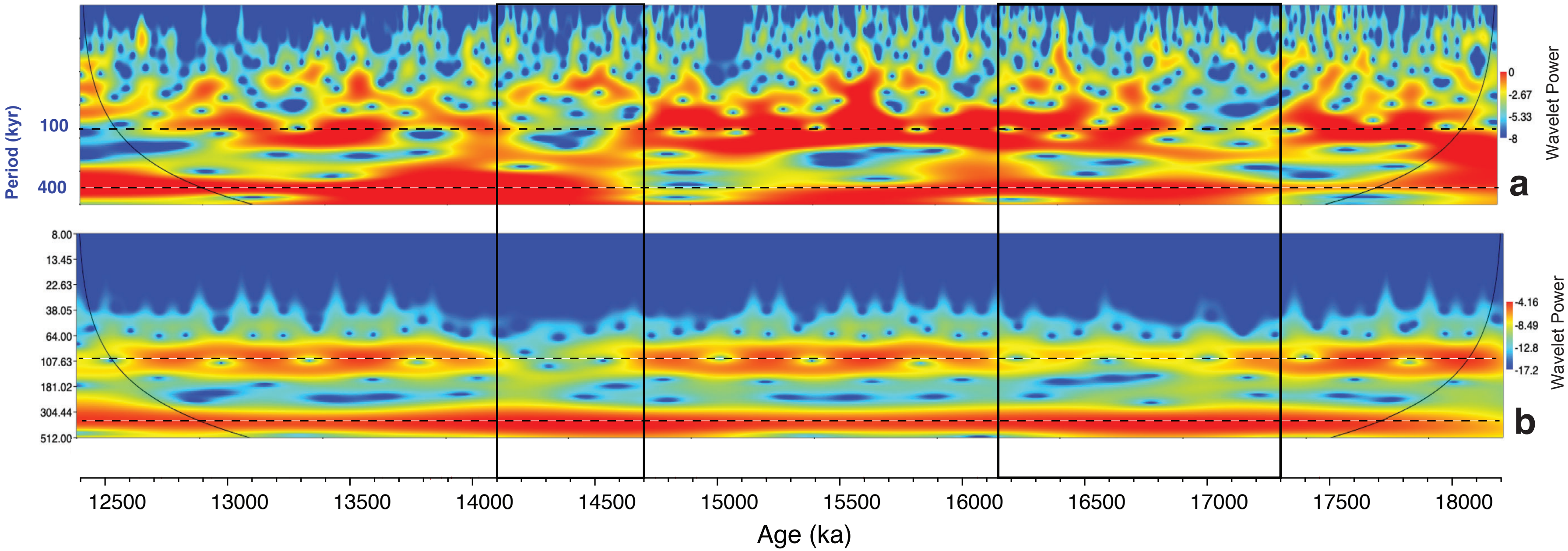

Figure S3-6. Wavelet power spectra of Site U1490 benthic foraminifer $\delta^{18}O$ (**a**) and eccentricity[7] (**b**). We note that weak 100 kyr eccentricity forcing between ~14.7 and 14.1 Ma and between ~17.3 and 16.1 Ma (marked by black rectangles) is reflected in the $\delta^{18}O$ time series. Black line indicates cone of influence; dotted lines indicate 100, 400 kyr periods.

SUPPLEMENTARY NOTE 4

Carbonate weight % estimates from calibrated X-ray fluorescence (XRF) scanner-derived elemental data

To calculate $CaCO_3$ weight % from the U1490 XRF scanner elemental data, we fitted a linear regression between the $CaCO_3$ measurements in 33 discrete samples (Supplementary Data File 4) and the average of three consecutive XRF scanner Log(Ca/(Al+Si)) values (corresponding to the 2 cm range of each discrete sample) (Figure S4-1, Supplementary Data File 5). X-ray fluorescence values at the top, centre and base of each discrete sample are plotted in Figure S4-2. The correlation between the $CaCO_3$ measurements and the XRF scanner elemental data improved, when logarithmic ratios of Ca against non-carbonate elements were used (Figure S4-1). The best correlation (R=0.92) was obtained, when normalizing Ca against the sum of Si and Al, which are typical elements of non-calcareous background sedimentation in the clay fraction and include biogenic opal. Regression lines and Pearson correlation coefficient did not show significant differences, when all three consecutive XRF scanner measurements were taken into account or the central measurement alone. We note that the lowest $CaCO_3$ value (82 weight %) at 14806 ka in Sample U1490A-25H-6, 46-48 cm corresponds to an unusually low XRF scanner Log(Ca/(Al+Si)) (Figure S4-2). We attribute the low values in $CaCO_3$ and Log(Ca/(Al+Si)) to the presence of a thin volcanic tephra layer at the base of the 2 cm-thick sample.

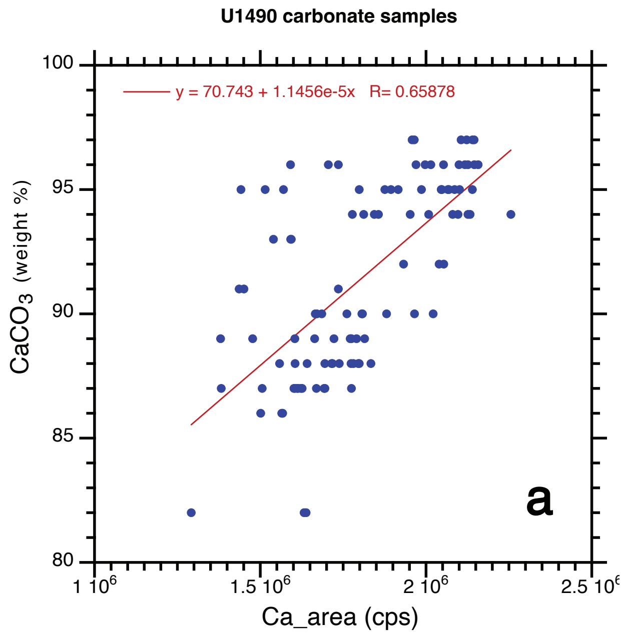

Figure S4-1. Evaluation of Site U1490 XRF scanner-derived Ca Log-ratios as proxy for $CaCO_3$ weight percentages. Regression plots of Ca_area (cps) in **a**, Log(Ca/(Al+Fe+K+Ti) in **b,** and Log(Ca/Al+Si) in **c** against carbonate concentration measurements of 33 discrete samples (2 cm thickness). Each discrete sample is plotted against three XRF scanner measurements (at 1 cm interval) corresponding to the top, centre and base of the sample.

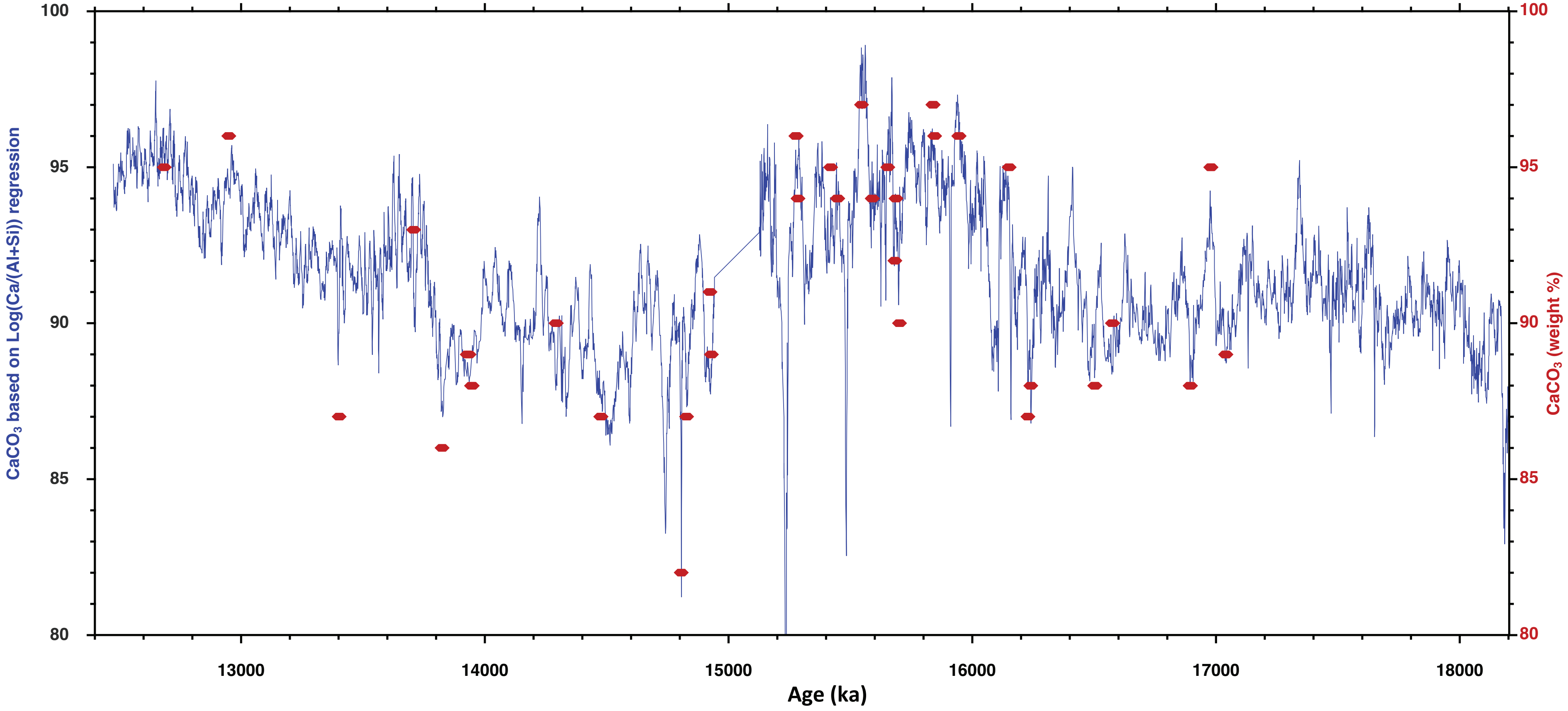

Figure S4-2. Comparison of XRF scanner-derived carbonate weight percentages (blue line) to carbonate concentration measurements of 33 discrete samples (red polygons) at Site U1490.

SUPPLEMENTARY NOTE 5

Carbonate accumulation, dissolution and carbonate ion concentration

We calculated the carbonate mass accumulation rate (MAR) by multiplying the bulk sediment MAR (product of sedimentation rate and dry bulk dry density of the sediment) with the proportion of $CaCO_3$ (% $CaCO_3$/100). The bulk density was taken from IODP Expedition 363 Gamma Ray Attenuation (GRA) shipboard measurements[5] transferred to the revised splice. Sedimentation rates were calculated using linear and locally weighted least squares (Stineman function) interpolation between the tie points of our tuned age model (Figure S5-1). We note that the smooth Stineman interpolation reduces the effect of artificial changes in sedimentation rates at tuning tie points. Both interpolations indicate relatively high carbonate accumulation rates during the interval 16 to 14.7 Ma, when coarse (>63 μm) fraction percentages of carbonate are low, indicating enhanced carbonate dissolution at the sea floor.

The relationship between in situ carbonate ion concentrations and >63 μm $CaCO_3$/total $CaCO_3$ has been shown to be constant in all major modern oceans with a slope for the regression line of 0.66 μmol/kg per % carbonate coarse fraction[28]. It was previously noticed that the reliability of the coarse fraction proxy is dependent on a relatively constant initial composition of the biogenic $CaCO_3$ and that it is less reliable for carbonate records with highly variable carbonate components such as Late Pleistocene glacial carbonate records[29]. This is due to the fact that the proportion of coarse size fraction may be biased by changes in the relative abundance of calcareous nannoplankton, which may determine the grain-size distribution more than the fragmentation of planktic foraminifers[30]. Moreover, coccoliths are significantly more resistant to dissolution than foraminifers and the ratio of coarse to fine $CaCO_3$ may, thus, be largely influenced by the extent of differential dissolution between coccoliths, dissolution resistant and dissolution susceptible foraminifers[31]. However, the composition of the carbonate fraction in the pelagic sediment at Site U1490 is relatively constant. Since coccoliths are the main component (80-90 %) of the bulk sediment at Site U1490[5], large changes in their abundance would result in major fluctuations in sedimentation rates, which are not evident (Figure S5-1).

We did not relate the variability in the >63 µm residue percentages to changes in bottom water current intensity, since the fine fraction<63 µm removed by winnowing would have resulted in reduced carbonate accumulation rates, which is not evident in our records (Figure S5-1). For example, carbonate accumulation rates were generally elevated between 13.8 and 13.6 Ma and between 17.4 and 17.8 Ma, when >63 µm residue percentages were high. The coarse (>63 μm) fraction percentage of carbonate has been successfully used to characterize carbonate dissolution and to track the distribution of carbonate ion undersaturated water masses over longer timescales in Neogene sediments with less dramatic glacial-interglacial variability of biogenic carbonate composition[32-35].

Figure S5-1 (next page). Site U1490 carbonate accumulation rates and coarse (>63 μm) fraction percentage of carbonate as proxies for carbonate dissolution and carbonate ion concentration, respectively. **a** Gamma ray attenuation (GRA) dry bulk density from IODP Expedition 363 shipboard measurements[5] transferred to the revised splice. **b** Carbonate content (weight %) from XRF Log(Ca/Terr.) measurements calibrated with discrete carbonate measurements (Supplementary Note 4). **c** Carbonate accumulation rates, calculated using linear interpolated sedimentation rates between tuning tie points with a constant dry bulk density of 1.6 g/cm^3. **d** Carbonate accumulation rates, calculated with a smooth curve fit to the tuning tie points using locally weighted least-squares (smooth Stineman function) and shipboard GRA dry bulk density values transferred to the revised splice. **e** >63 μm fraction (weight % of bulk sediment). **f** >63 μm weight % of carbonate as proxy for carbonate ion in-situ saturation[28].

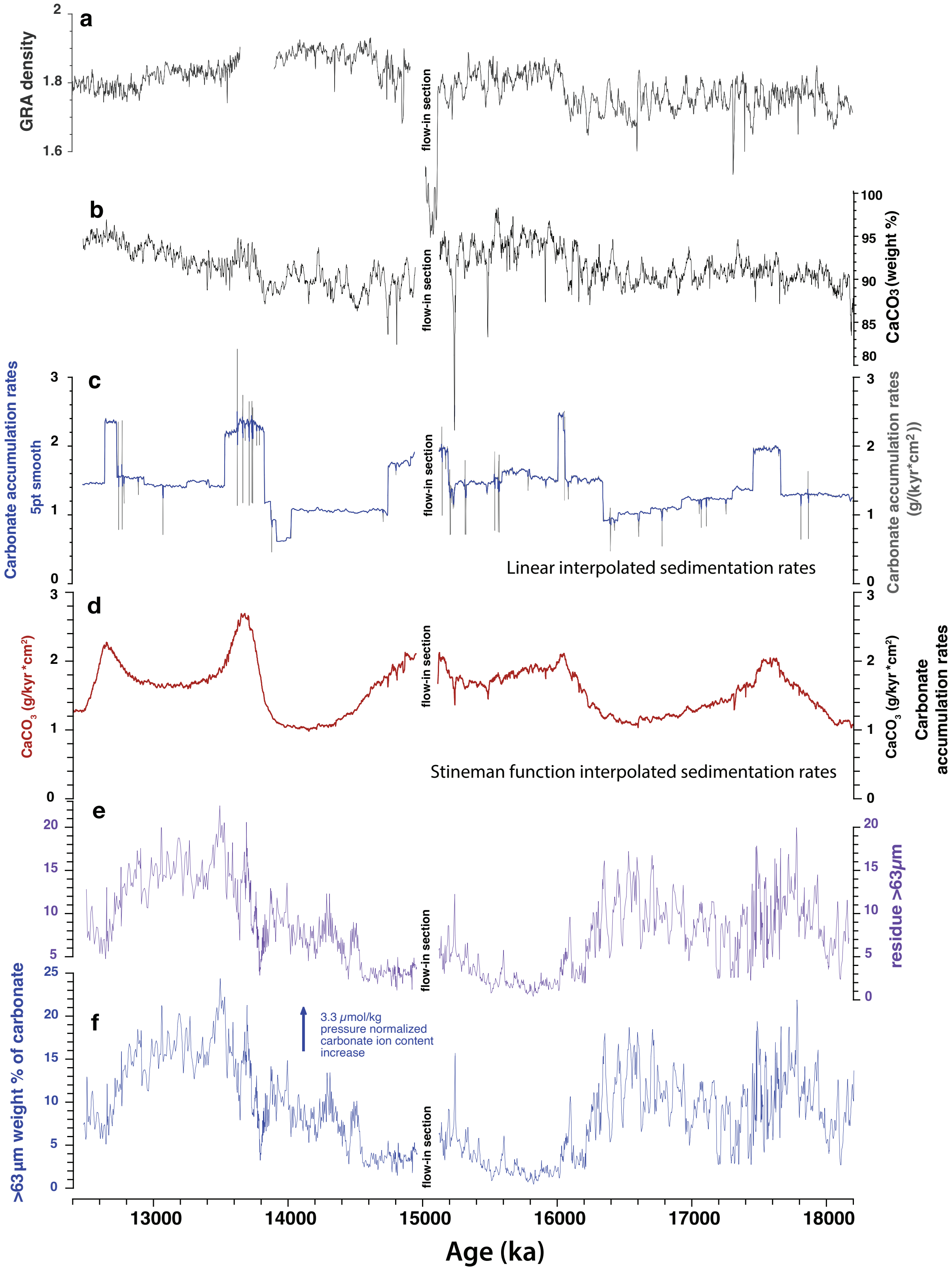
a
GRA density
b
CaCO3 (weight %)
c
Carbonate accumulation rates
5pt smooth
Carbonate accumulation rates (g/(kyr*cm²))
Linear interpolated sedimentation rates
d
CaCO3 (g/kyr*cm²)
Carbonate accumulation rates
Stineman function interpolated sedimentation rates
e
residue >63µm
f
>63 µm weight % of carbonate
3.3 µmol/kg pressure normalized carbonate ion content increase
flow-in section
Age (ka)

Figure S5-1

During the initial, warmer phase of the MCO (~16.9-16 Ma), U1490 coarse (>63 μm) fraction weight% of carbonate shows high coherence (~0.8) and a distinctive antiphase relationship to benthic foraminifer $\delta^{18}O$ in response to 100 kyr eccentricity and 41 kyr obliquity forcing (Figure S5-2). The cross spectral analysis for the interval 17 to 16 Ma indicates that 100 kyr- and 41 kyr-paced warmer intervals (low $\delta^{18}O$ values) are characterized by improved carbonate preservation, as shown by high coarse >63 μm fraction weight% percentages (Figure S5-2).

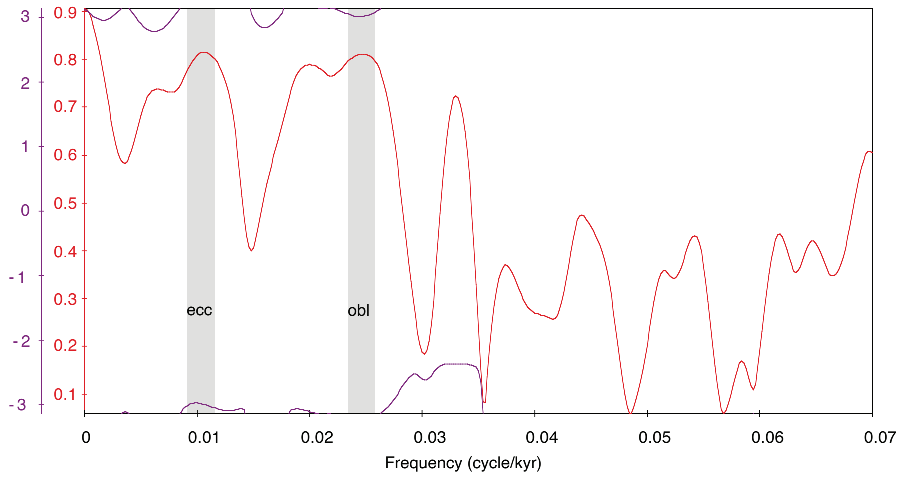

Figure S5-2. Phase relationship between Site U1490 >63 μm carbonate coarse fraction and benthic foraminifer $\delta^{18}O$ between 17 and 16 Ma. Blackman Tukey cross spectrum of coherence and phase was performed in AnalySeries 2.08[9] using a Bartlett window, bandwidth is 0.005, non-zero coherence is higher than 0.38. Red scale is coherence, magenta scale is phase in radians. Note consistent antiphase relationship (high >63 μm weight% at low $\delta^{18}O$) at the short eccentricity (ecc) and obliquity (obl) bands (grey shaded areas).

SUPPLEMENTARY NOTE 6

Variability of XRF scanner-derived Log(Si/Ti) as proxy for biogenic silica

X-ray fluorescence scanner-derived Log(Si/Ti) has been previously used to evaluate the biogenic Si content of Miocene pelagic sediments in the eastern equatorial Pacific Ocean[36-38]. This approach assumed a constant Si/Ti ratio in the terrigenous input (dust and fine grained current transported clay). Terrigenous sedimentation at Site U1490 displays minimal grain-size and compositional variability as a consequence of the site's distal depositional environment on a pelagic ridge. Since Ti is a consistent component of clay minerals that is hardly affected by chemical weathering and diagenesis, the variability in Log(Si/Ti) at Site U1490 is mainly driven by the variability in Si, as a result of surplus deposition of biogenic opal. Visual examination of washed residues revealed common radiolarian tests in the 63-150 μm fractions of samples with high XRF scanner Log(Si/Ti) values. We, thus, interpret high Log(Si/Ti) values as an indicator of elevated biogenic silica content, reflecting both higher productivity of siliceous microfossils and better preservation of biogenic silica due to Si-enriched deep water.

A distinctive feature of the Site U1490 Log(Si/Ti) record is its antiphase co-variation with benthic foraminifer δ^{18}O, which is expressed both in the long-term trends and in astronomical-scale variations during the MCO, in particular between ~16.9 and 16 Ma (Figure 3, Figure S6-1). High Log(Si/Ti) values characterize warm (low δ^{18}O) intervals at eccentricity maxima during this warmer interval of the MCO (Figure S6-1). This phase relationship is evident in the power spectrum of Log(Si/Ti) and in the coherence and phase between Log(Si/Ti) and δ^{18}O for the time interval between 17 and 15.2 Ma (Figure S6-2). Log(Si/Ti) exhibits high spectral power in the 100 kyr eccentricity band (> 99% confidence), which displays the same double peak at the 95 and 125 kyr wavelength as eccentricity as well as a peak at the 41 kyr obliquity band (>95 % confidence). The same prominent spectral peaks are displayed in the Blackman-Tukey cross spectral analysis with δ^{18}O, which shows coherence k = 0.9 at the eccentricity and k = 0.8 at the obliquity band (Figure S6-2b). A similar coherence and phase relationship at the eccentricity and obliquity bands are evident in the cross spectra between Log(Si/Ti) and weight % of carbonate (coherence k = 0.8 at the eccentricity band and 0.75 at the obliquity band) (Figure S6-3).

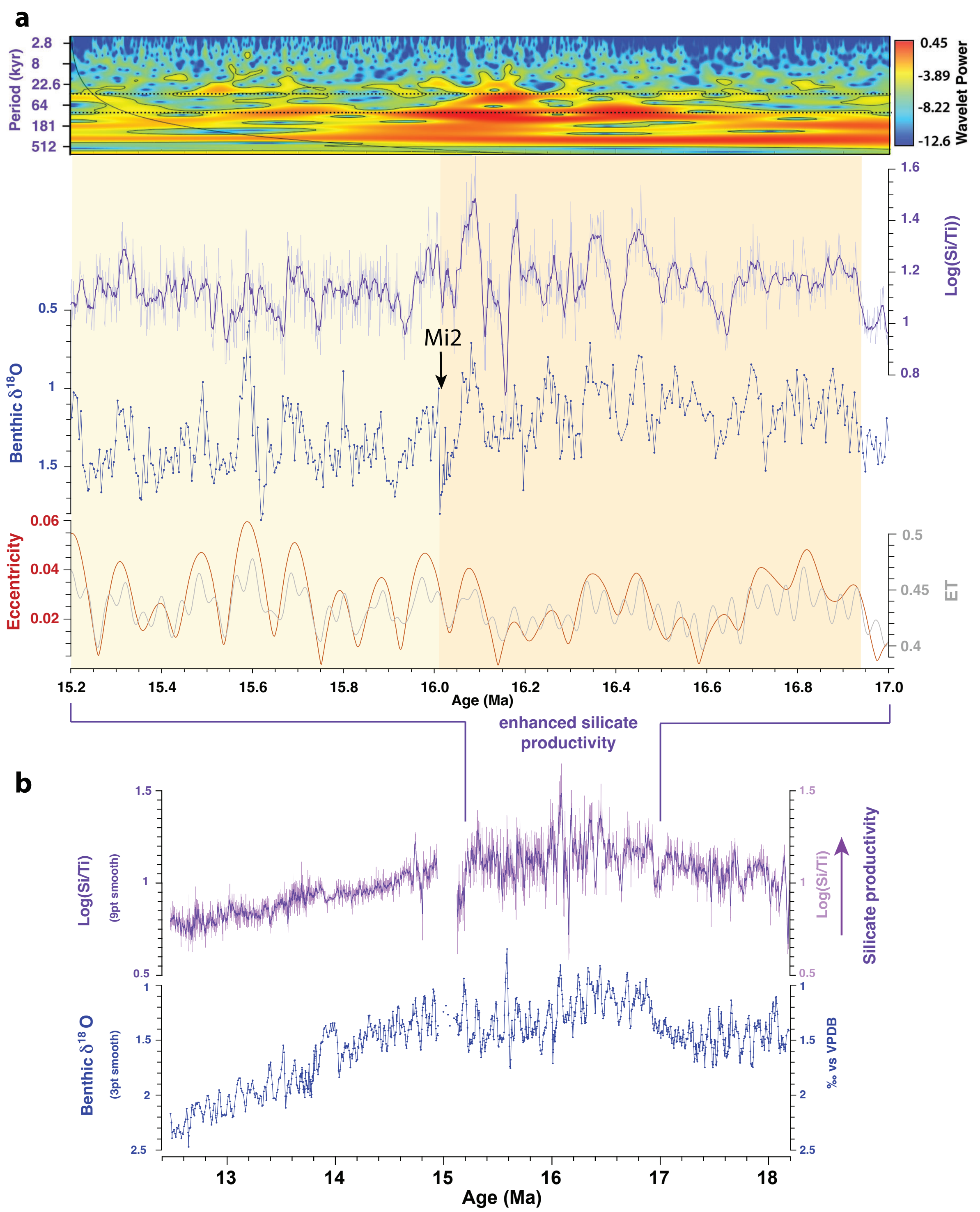

Figure S6-1. Evolution of XRF scanner-derived Log(Si/Ti) at Site U1490. **a** Wavelet power spectrum of Log(Si/Ti) in comparison to benthic foraminifer $\delta^{18}O$ and orbital configuration. Maxima in Log(Si/Ti) coincide with $\delta^{18}O$ minima, indicating increased opal concentrations during warm intervals associated with eccentricity and obliquity maxima between 17 and 15.2 Ma. Note dominance of 41 kyr (obliquity) and 100 kyr (eccentricity) periodicities (indicated by dashed black lines in wavelet spectrum) and change in amplitude variability across Mi2 transition (~16 Ma). Warmer phase of MCO from ~16.9 to 16 Ma shaded dark orange; hyperthermal phase of MCO following Mi2 glaciation shaded light orange. **b** Long-term trends in silicate productivity (9 point moving average) also follow benthic foraminifer $\delta^{18}O$ (3 point moving average) between 18.2 and 12.5 Ma.

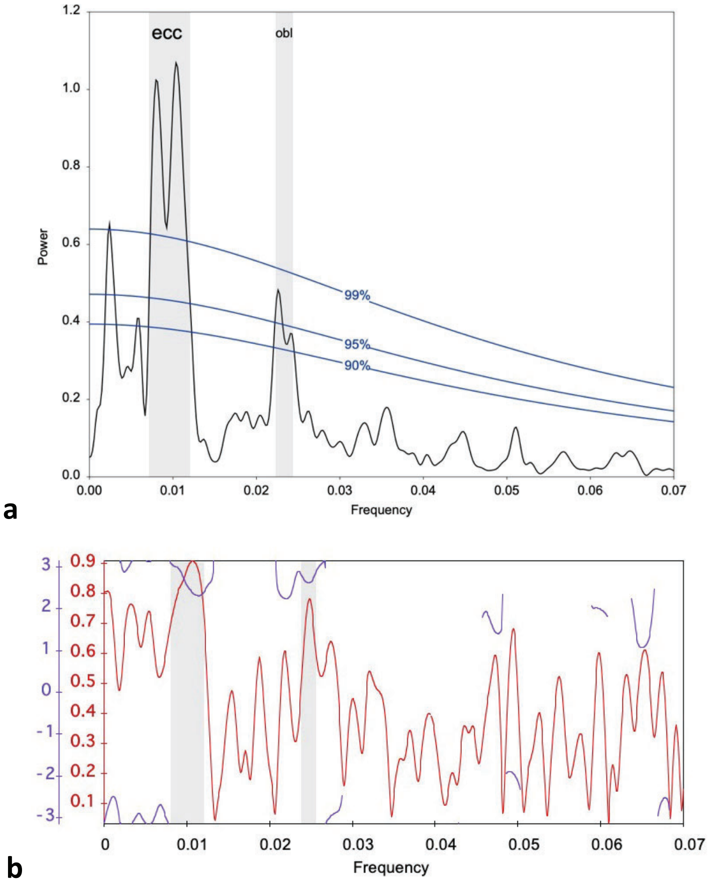

Figure S6-2. Phase relationship between Site U1490 Log(Si/Ti) and benthic foraminifer $\delta^{18}O$ over the interval 17 to 15.2 Ma. **a** REDFIT power spectrum[27] of Log(Si/Ti); confidence intervals (chi^2) of 99 %, 95 % and 90 % are given as blue lines. **b** Blackman Tukey cross spectrum of coherence and phase[9] using a Bartlett window; bandwidth is 0.0028, non-zero coherence is higher than 0.38. Red scale is coherence, blue scale phase in radians. Note consistent antiphase relationship (high silica at low $\delta^{18}O$) at the short eccentricity (ecc) and obliquity (obl) bands (gray shaded areas).

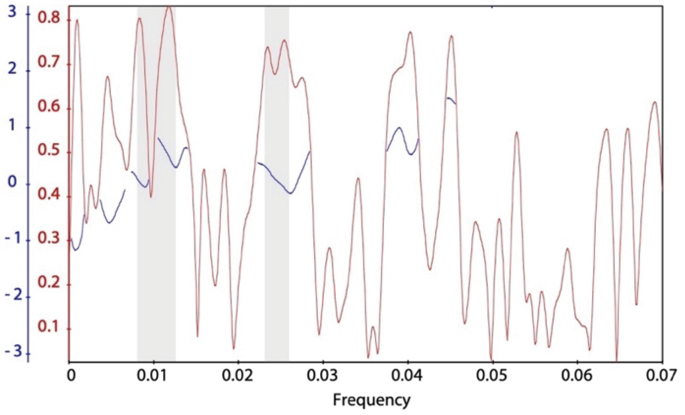

Figure S6-3. Phase relationship between Site U1490 Log(Si/Ti) and >63 μm weight% of carbonate as proxy of carbonate dissolution / carbonate ion concentration over the interval 17 to 15.2 Ma. Blackman Tukey cross spectrum of coherence and phase[9] using a Bartlett window, bandwidth is 0.0028, non-zero coherence is higher than 0.38. Red scale is coherence, blue scale phase in radians. Note consistent in-phase relationship (high silicon at high weight % of carbonate (= high carbonate concentration, low dissolution) at the short eccentricity and obliquity bands (grey shaded areas).

SUPPLEMENTARY NOTE 7

Comparison of stable isotope records from Pacific and Indian Oceans

The paleo-locations of sites referred to in this study are shown in Figure S7-1 and listed in Table S7-1. The composition of benthic foraminifer assemblages at Site U1490 suggests that the late Early to Middle Miocene water depth at this location was close to that of today (lower bathyal). This is also supported by benthic foraminifer $\delta^{18}O$ values, which are close to those of sites with a comparable water depth range (ODP Sites 761, 1171 and 1237) during the late Early to Middle Miocene (Figure S7-2). Comparison of benthic benthic foraminifer $\delta^{18}O$ and $\delta^{13}C$ records from the Southern, Indian and Pacific Oceans are shown in Figures S7-2 and S7-3.

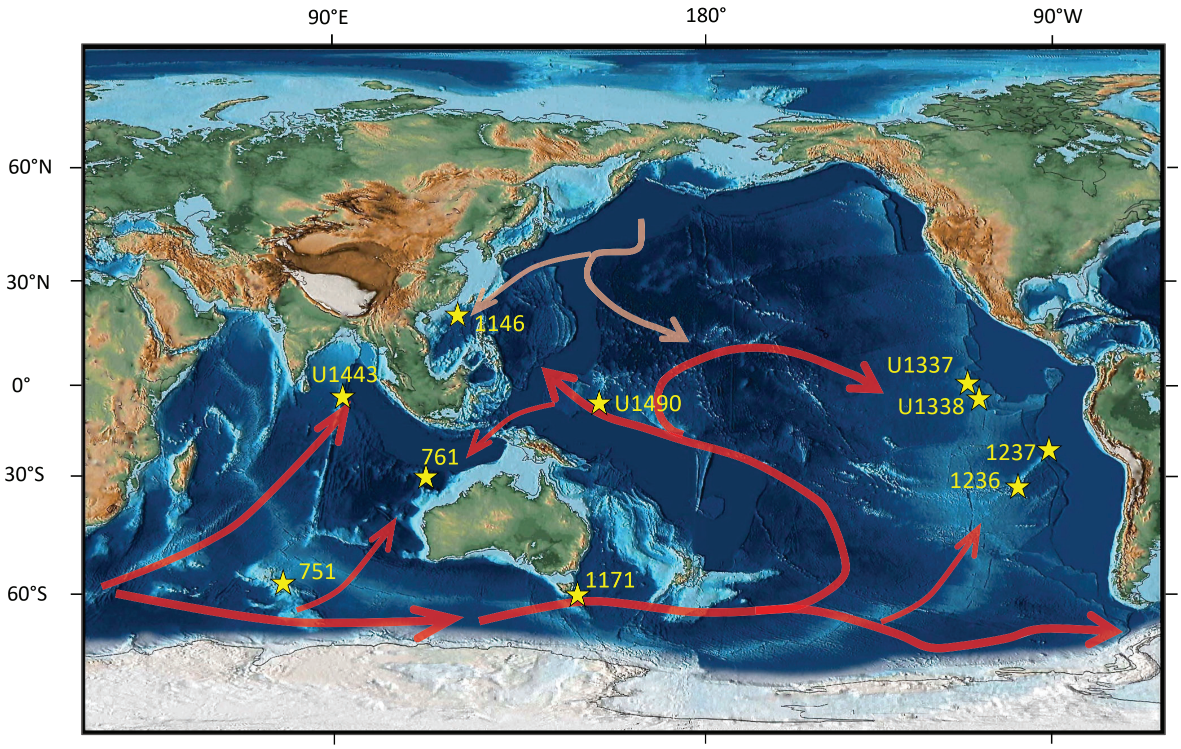

Figure S7-1. Hypothetical Indian and Pacific Ocean deep water circulation paths during the MCO with paleo-positions of sites referred to in this study. Paleogeographic reconstruction for 15 Ma from PALEOMAP PaleoAtlas for GPlates[39].

Table S7-1. Modern location, backtracked position, paleo-water depth and data source for ODP and IODP sites referred to in this study. BF = benthic foraminifers, mbsl = meters below sea level.

Site	Latitude	Longitude	Backtracked position	Modern water depth	Miocene water depth	Data source
**U1490**	05°48.95'N	142°39.27'E	Paleo-latitude: 3-4°N; Paleo-longitude: no marked change	2341 mbsl	close to present based on BF assemblage composition and comparison of BF $\delta^{18}O$	Ref. 5, this work Paleolatitude.org[40]
**U1337**	3°50.009'N	123°12.352'W	Paleo-latitude: ±1° of Equator; Paleo-longitude: 110-118°W	4463 mbsl	3800-4100 mbsl (18-13 Ma)	Refs. 13-15, 41-42
**U1138**	2°30.469'N	117°58.178'W	Paleo-latitude: 0-2°S; Paleo-longitude: 104-112°W	4200 mbsl	3400-3700 mbsl (16-13 Ma)	Refs. 13-15, 41-42
**U1443**	5° 23.01'N	90° 21.7'E)	Paleo-latitude: 0-2°S; Paleo-longitude: no marked change	2925 mbsl	2500-3000 mbsl	Refs. 13-14, 16, 43 Paleolatitude.org[40]
**1237**	16°0.421'S	76°22.685'W	Paleo-latitude: ~20°S; Paleo-longitude: 10° westward of present position	3212 mbsl	2500-3000 mbsl	Refs. 13-14, 17-18, 44
**1236**	21°21.539'S	81°26.165'W	Paleo-latitude: ~25°S; Paleo-longitude: ~10° westward of present position	1323 mbsl	500-1000 mbsl	Refs. 13-14, 18, 44
**1171**	48°30'S	149°06.69'E	Paleo-latitude: 55°S; Paleo-longitude: no marked change	2150 mbsl	1600 mbsl	Refs. 19-20, 45
**1146**	19°27.40'N	116°16.37'E	Paleo-latitude: ~23°N; Paleo-longitude: no marked change	2091 mbsl	close to present based on BF	Refs. 13-14, 17, 20-21 Paleolatitude.org[40]
**761**	16°44'S	115°32'E	Paleo-latitude: ~25°S; Paleo-longitude: no marked change	2168 mbsl	close to present based on BF	Ref. 22 Paleolatitude.org[40]
**751**	57°43.56'S	79°48.89'E	Paleo-latitude: 56°S; Paleo-longitude: no marked change	1634 mbsl	1500-1600 mbsl	Refs. 11, 13-14, 46 Paleolatitude.org[40]

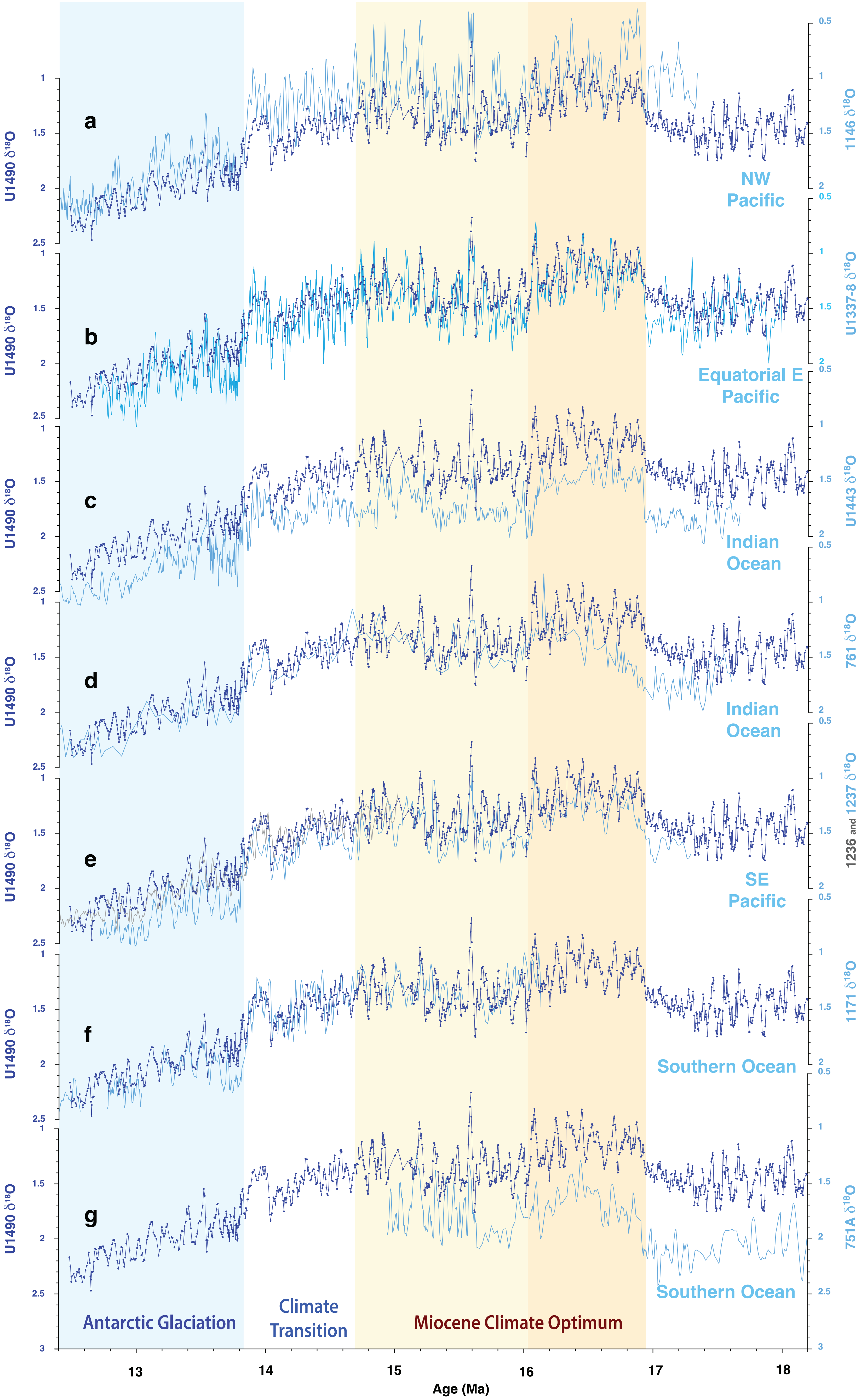

Figure S7-2. Comparison of Site U1490 benthic foraminifer $\delta^{18}O$ record to astronomically-tuned Southern, Indian and Pacific Ocean records (Table S7-1). All records are given in ‰ vs. VPDB and as 3 point moving averages (except for Site 761). Warmer phase of MCO from ~16.9 to 16 Ma shaded dark orange; hyperthermal phase of MCO following Mi2 glaciation shaded light orange. Expansion of Antarctic ice sheet following Mi3 shaded light blue. Data source of isotope records is given in Supplementary Note 2 and in Supplementary Data Files 1, 6, 7, 8, 10-14.

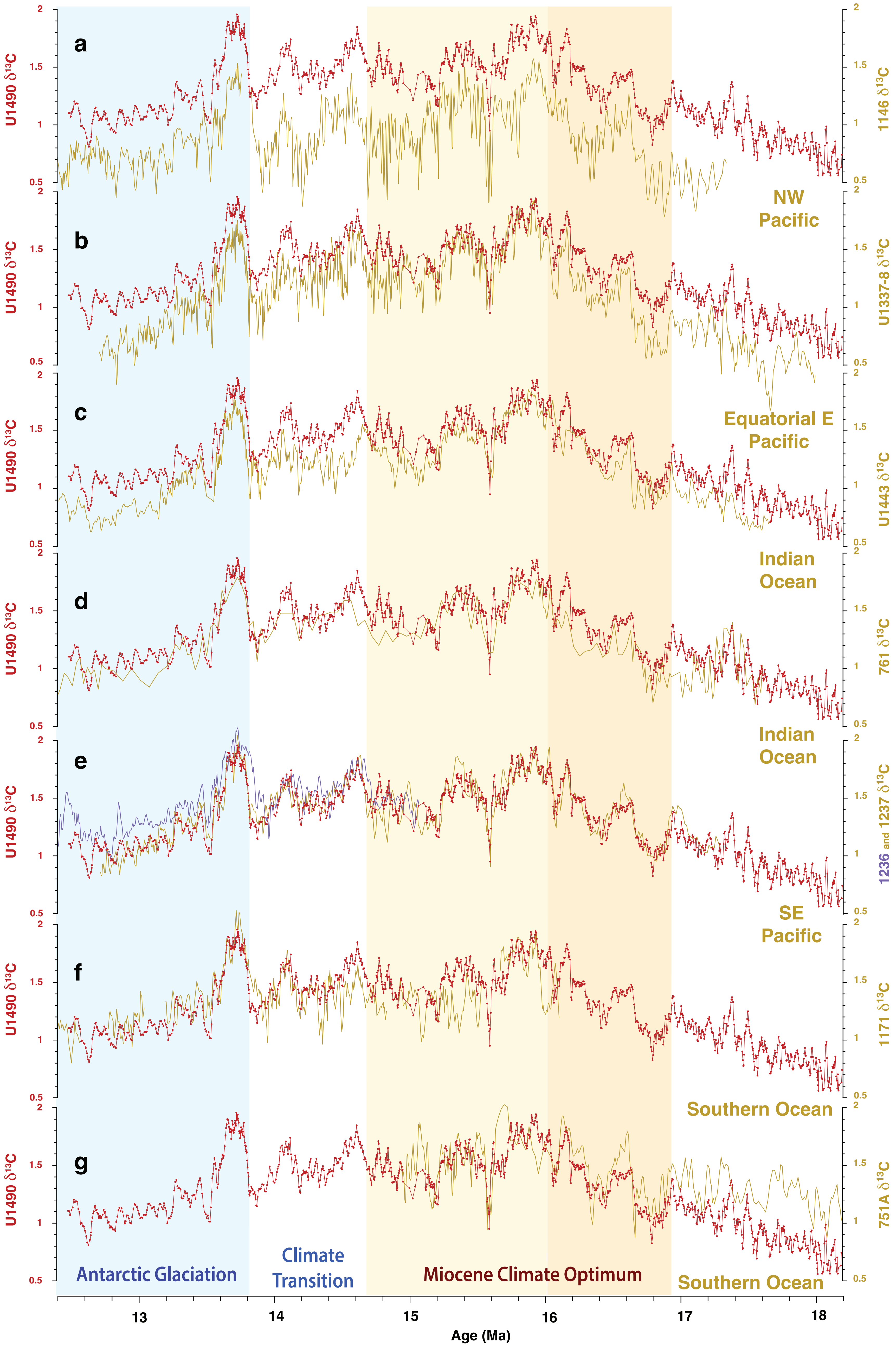

Figure S7-3. Comparison of Site U1490 benthic foraminifer $\delta^{13}C$ record to astronomically-tuned Southern, Indian and Pacific Ocean records (Table S7-1). All records are given in ‰ vs. VPDB and are given as 3 point moving averages (except for Site 761). Warmer phase of MCO from ~16.9 to 16 Ma shaded dark orange; hyperthermal phase of MCO following Mi2 glaciation shaded light orange. Expansion of Antarctic ice sheet following Mi3 shaded light blue. Data source of isotope records is given in Supplementary Note 2 and in Supplementary Data Files 1, 6, 7, 8, 10-14.

SUPPLEMENTARY REFERENCES

1. Kawabe, M. & Fujio, S. Pacific Ocean circulation based on observation. *J. Oceanogr.* 66, 389–403 (2010). https://doi.org/10.1007/s10872-010-0034-8

2. Talley, L.D. Closure of the global overturning circulation through the Indian, Pacific, and Southern Oceans: Schematics and transports. *Oceanography* 26(1), 80–97 (2013). https://doi.org/10.5670/oceanog.2013.07

3. Sigman, D.M. *et al.* The Southern Ocean during the ice ages: A review of the Antarctic surface isolation hypothesis, with comparison to the North Pacific. *Quat. Sci. Rev.* 254, 106732 (2021). https://doi.org/10.1016/j.quascirev.2020.106732

4. Talley, L.D. Hydrographic Atlas of the World Ocean Circulation Experiment (WOCE), Vol. 2, Pacific Ocean (WOCE International Project Office, Southampton, U.K. (2007).

5. Rosenthal, Y. et al. Western Pacific warm pool. In *Proceedings of the International Ocean Discovery Program, Vol. 363* (IODP, College Station, 2018). https://doi.org/10.14379/iodp.proc.363.2018

6. Wilkens, R.H., Westerhold, T., Drury, A.J., Lyle, M., Gorgas, T., and Tian, J. Revisiting the Ceara Rise, equatorial Atlantic Ocean: isotope stratigraphy of ODP Leg 154 from 0 to 5 Ma. *Clim. Past* 13, 779–793 (2017). https://doi.org/10.5194/cp-13-779-2017

7. Laskar, J. *et al.* A long-term numerical solution for the insolation quantities of the Earth. *Astron. Astrophys.* 428, 261–285 (2004). https://doi.org/10.1051/0004-6361:20041335

8. Holbourn, A.E., Kuhnt, W., Schulz, M. & Erlenkeuser, H. Impacts of orbital forcing and atmospheric carbon dioxide on Miocene ice-sheet expansion. Nature 438, 483–487 (2005).

9. Paillard, D., Labeyrie, L. & Yiou, P. Macintosh Program performs time-series analysis. *Eos, Transactions AGU* 77, 379–379 (1996). https://doi.org/ 10.1029/96EO00259

10. Miller, K.G., Wright, J.D. & Fairbanks, R.G. Unlocking the ice house: Oligocene–Miocene oxygen isotopes, eustasy and margin erosion. *J. Geophys. Res.* 96, 6829–6848 (1991). https://doi.org/10.1029/90JB02015

11. Miller, K.G., Baluyot, R., Wright, J.D., Kopp, R.E. & Browning, J.V. Closing an early Miocene astronomical gap with Southern Ocean $\delta^{18}O$ and $\delta^{13}C$ records: Implications for sea level change. *Paleoceanography* 32, 600–621 (2017). https://doi.org/10.1002/2016PA003074

12. Miller K.G. *et al.* Cenozoic sea-level and cryospheric evolution from deep-sea geochemical and continental margin records. *Sci. Adv.* 6, eaaz1346 (2020). https://doi.org/10.1126/sciadv.aaz1346

13. Holbourn, A.E., Kuhnt, W., Kochhann, K.G.D., Matsuzaki, K.M. & Andersen, N. Middle Miocene Climate-Carbon Cycle Dynamics: Keys for Understanding Future Trends on a Warmer Earth?. PANGAEA https://doi.org/10.1594/PANGAEA.922280 (2020)

14. Holbourn, A.E., Kuhnt, W., Kochhann, K.G.D., Matsuzaki, K.M. & Andersen, N. Middle Miocene climate-carbon cycle dynamics: Keys for understanding future trends on a warmer Earth? In *Understanding the Monterey Formation and similar biosiliceous units across space and time* (eds. Aiello, I., Barron, J. & Ravelo, C.), *Geol. Soc. Am. Special Paper* 556, 1–19 (2022). https://doi.org/10.1130/2022.2556(05)

15. Wilkens, R.H., Dickens, G.R., Tian, J., Backman, J. & Expedition 320/321 Scientists Data report: Revised composite depth scales for Sites U1336, U1337 and U1338. In (eds. Pälike, H. et al.) *Proceedings of the IODP* 320/321, 1–158, Integrated Ocean Drill. Program Manage. Int., Inc., Tokyo (2013). doi:10.2204/iodp.proc.320321.209.2013.

16. Kochhann, K.G. *et al.* Impacts of the Middle Miocene Climatic Transition on deep-water carbonate preservation and oxygenation in the equatorial Indian Ocean. *Palaeogeogr. Palaeoclimatol. Palaeoecol.* 576, 110511 (2021). https://doi.org/10.1016/j.palaeo.2021.110511

17. Holbourn, A.E., Kuhnt, W., Schulz, M., Flores, J.-A., & Andersen, N. Orbitally-paced climate evolution during the middle Miocene "Monterey" carbon-isotope excursion. *Earth Planet. Sci. Lett.* 261, 534–550 (2007). https://doi.org/10.1016/j.epsl.2007.07.026

18. Holbourn, A.E., Kuhnt, W., Frank, M. & Haley, B.A. Changes in Pacific Ocean circulation following the Miocene onset of permanent Antarctic ice cover. *Earth Planet. Sci. Lett.* 365, 38–50 (2013). https://doi.org/10.1016/j.epsl.2013.01.020

19. Shevenell, A.E. & Kennett, J.P. Paleoceanographic change during the middle Miocene climate revolution: an Antarctic stable isotope perspective. In *The Cenozoic Southern Ocean: Tectonics, Sedimentation, and Climate Change Between Australia and Antarctica* Vol. 151 (eds. Exon, N., Kennett, J.P. & Malone, M.), 235–252 (AGU, Washington, D. C., 2004a).

20. Shevenell, A.E., Kennett, J.P. & Lea, D.W. Middle Miocene Southern Ocean Cooling and Antarctic Cryosphere expansion. *Science* 305, 1766–1770 (2004b). https://doi.org/10.1126/science.1100061

21. Holbourn, A., Kuhnt, W., Clemens, S.C., & Heslop, D.A ~12 Myr Miocene record of East Asian Monsoon variability from the South China Sea. *Paleoceanography and Paleoclimatology* 36, (2021). e2021PA004267. https://doi.org/10.1029/2021PA00426722.

22. Holbourn, A.E., Kuhnt, W., Simo, J.A. & Li, Q. Middle Miocene isotope stratigraphy and paleoceanographic evolution of the northwest and southwest Australian margins (Wombat Plateau and Great Australian Bight). *Palaeogeogr. Palaeoclimatol. Palaeoecol.* 208, 1–22 (2004). https://doi.org/10.1016/j.palaeo.2004.02.003

23. Ogg, J.G. Chapter 5 Geomagnetic Polarity Time Scale. In: *The Geologic Time Scale 2012* Vol. 1 (eds. Gradstein, F.M., Ogg, J.G., Schmitz, M.D., & Ogg, G.M.), 85–113 (Elsevier, 2012). https://doi.org/10.1016/B978-0-444-59425-9.00005-6

24. Ogg, J.G. Chapter 5 Geomagnetic Polarity Time Scale. In: *The Geologic Time Scale 2020* Vol. 1 (eds. Gradstein, F.M., Ogg, J.G., Schmitz, M.D. & Ogg, G.M), 159–191 (Elsevier, 2020). https://doi.org/10.1016/B978-0-12-824360-2.00005-X

25. Kasbohm, J. *et al.* Eruption history of the Columbia River Basalt Group constrained by high-precision U-Pb and 40Ar/39Ar geochronology. *Earth Planet. Sci. Lett.* 617, 118269 (2023). https://doi.org/10.1016/j.epsl.2023.118269

26. Schulz, M. & Mudelsee, M. REDFIT: estimating red-noise spectra directly from unevenly spaced paleoclimatic time series. *Comput. Geosci.* 28, 421–426 (2002). https://doi.org/10.1016/S0098-3004(01)00044-9

27. Hammer, Ø., Harper, D.A.T. & Ryan, P.D. PAST: paleontological statistics software package for education and data analysis. *Palaeontol. Electronica* 4, 1–9 (2001).

28. Broecker, W.S. *et al.* Evidence for a reduction in the carbonate ion content of the deep sea during the course of the Holocene. *Paleoceanography* 14, 744–752 (1999). https://doi.org/10.1029/1999PA900038

29. Broecker, W. & Clark E. Ratio of coccolith $CaCO_3$ to foraminifera $CaCO_3$ in late Holocene deep sea sediments. *Paleoceanography* 24, PA3205 (2009). https://doi.org/10.1029/2009PA001731

30. Hancock, H.J.L. & Dickens, G.R. Carbonate dissolution episodes in Paleocene and Eocene sediment, Shatsky Rise, west-central Pacific. In *Proceedings of the Ocean Drilling Program, Scientific Results* Vol. 198 (eds. Bralower, T.J., Premoli Silva, I., & Malone, M.J.), 1–24, College Station, TX (Ocean Drilling Program, 2005) http://wwwodp.tamu.edu/publications/198_SR/volume/chapters/116

31. Chiu, T.-C. & Broecker, W.S. Toward better paleocarbonate ion reconstructions: New insights regarding the $CaCO_3$ size index. *Paleoceanography* 23, PA2216 (2008). https://doi.org/10.1029/ 2008PA001599

32 Roth, J.M., Droxler, A.W. & Kameo, K. The Caribbean carbonate crash at the middle to late Miocene transition: Linkage to the establishment of the modern global ocean conveyor. In *Proceedings of the Ocean Drilling Program, Scientific Results* Vol. 165 (eds. Leckie, R.M., Sigurdsson, H., Acton, G.D. & Draper, G.), College Station, TX (Ocean Drilling Program, 2000). https://doi.org/10.2973/odp.proc.sr.165.013.2000

33. Kastanja, M.M. & Henrich, R. Grain-size variations in pelagic carbonate oozes from the Walvis Ridge–SE Atlantic Ocean (ODP Site 1265): A low resolution Miocene record of carbonate sedimentation and preservation. *Mar. Geol.* 237, 97–108 (2007). https://doi.org/10.1016/j.margeo.2006.10.040

34. Preiß-Daimler, I.V., Henrich, R., & Bickert, T. The final Miocene carbonate crash in the Atlantic: Assessing carbonate accumulation, preservation and production. *Mar. Geol.* 343, 39–46 (2013). http://doi.org/10.1016/j.margeo.2013.06.010

35. Lübbers, J. *et al.* The middle to late Miocene "Carbonate Crash" in the equatorial Indian Ocean. *Paleoceanogr. Paleoclimatol.* 34, 813–832 (2019). https://doi.org/10.1029/2018PA003482

36. Holbourn, A. *et al.* Middle Miocene climate cooling linked to intensification of eastern equatorial Pacific upwelling. *Geology* 42, 19–22 (2014). https://doi.org/10.1130/G34890.1

37. Lyle, M. & Baldauf, J. Biogenic sediment regimes in the Neogene equatorial Pacific, IODP Site U1338: Burial, production, and diatom community. *Palaeogeogr. Palaeoclimatol. Palaeoecol.* 433, 106–128 (2015). https://doi.org/10.1016/j.palaeo.2015.04.001

38. Lyle, M., Drury, A.J., Tian, J., Wilkens, R. & Westerhold, T. Late Miocene to Holocene high-resolution eastern equatorial Pacific carbonate records: stratigraphy linked by dissolution and paleoproductivity. *Clim. Past* 15, 1715–1739 (2019).

39. Scotese, C.R. PALEOMAP PaleoAtlas for GPlates and the Paleo- Data Plotter Program. PALEOMAP Project, doi:10.13140/RG2.2.34367.00166.

40. van Hinsbergen, D.J. *et al.* A paleolatitude calculator for paleoclimate studies. *PLoS One* 10, e0126946 (2015). https://doi.org/10.1371/journal.pone.0126946

41. Pälike, H. *et al.* Expedition 320/321 summary. In *Proceedings of the Integrated Ocean Drilling Program, Volume 320/321* (eds. Pälike, H. *et al.*), 1–141 (IODP-MI, Tokyo, 2010). https://doi.org/10.2204/iodp.proc.320321.101.2010

42. Pälike, H. *et al.* A Cenozoic record of the equatorial Pacific carbonate compensation depth. *Nature* 488, 609–614 (2012). https://doi.org/10.1038/ nature11360

43. Zachos, J.C., Rea, D.K., Seto, K., Niitsuma, N. & Nomura, R. Paleogene and early Neogene deep water history of the Indian Ocean: inferences from stable isotopic records. In Synthesis of Results from Scientific Drilling in the Indian Ocean (eds. Duncan, R.A., Rea, D.K., Kidd, R.B., von Rad, U. & Weissel, J.K.). *AGU, Geophys. Monogr.* 70, 351–386. https://doi.org/10.1029/GM070p0351

44. Mix, A.C. *et al.* Proceeding of the Ocean Drilling Program, Initial Reports Vol. 202, College Station, TX (Ocean Drilling Program, 2003). https://doi.org/10.2973/odp.proc.ir.202.2003

45. Shevenell, A.E., Kennett, J.P., and Lea, D.W. Middle Miocene ice sheet dynamics, deep-sea temperatures, and carbon cycling: A Southern Ocean perspective: Geochemistry, Geophysics, Geosystems, 9, Q02006 (2008). https://doi.org/10.1029/2007GC001736

46. Wright, J.D., Miller, K.G. & Fairbanks, R.G. Early and Middle Miocene stable isotopes: Implications for Deepwater circulation and climate. *Paleoceanogr. Paleoclimatol.* 7, 357–389 (1992). https://doi.org/10.1029/92PA0076041
