# Supplementary material for: Re-organization of Pacific overturning circulation across the Miocene Climate Optimum
Source: Nat Commun. 2024 Sep 17;15:8135. doi: 10.1038/s41467-024-52516-x (PMC11408672; doi:10.1038/s41467-024-52516-x)
Supplement: Supplementary file 3 — Description of Additional Supplementary Files [file 41467_2024_52516_MOESM3_ESM.pdf]

Supplementary Data File 1. U1490 stable isotopes and coarse fraction residue
Supplementary Data File 2. U1490 stable isotope offsets *C. mundulus - Rectuvigerina*
Supplementary Data File 3. U1490 stable isotope offsets *C. mundulus - O. umbonatus*
Supplementary Data File 4. U1490 carbonate measurements
Supplementary Data File 5. U1490 XRF-scanner elemental data and XRF-derived $CaCO_3$ weight %
Supplementary Data File 6. U1338-U1337 stable isotopes on revised age model
Supplementary Data File 7. 1146 revised splice, age model and stable isotopes
Supplementary Data File 8. 761B stable isotopes on revised age model
Supplementary Data File 9. CO_2 data from Rae et al. (2021) including revised 761B ages
Supplementary Data File 10. 751A stable isotopes on revised age model
Supplementary Data File 11. 1236 stable isotopes on revised age model
Supplementary Data File 12. 1237 stable isotopes on revised age model
Supplementary Data File 13. U1443 stable isotopes
Supplementary Data File 14. 1171C stable isotopes on revised age model
